# Prefrontal Responses during Proactive and Reactive Inhibition Are Differentially Impacted by Stress in Anorexia and Bulimia Nervosa

**DOI:** 10.1523/JNEUROSCI.2853-20.2021

**Published:** 2021-05-19

**Authors:** Margaret L. Westwater, Flavia Mancini, Adam X. Gorka, Jane Shapleske, Jaco Serfontein, Christian Grillon, Monique Ernst, Hisham Ziauddeen, Paul C. Fletcher

**Affiliations:** ^1^Department of Psychiatry, Addenbrooke's Hospital, University of Cambridge, Cambridge CB2 0SZ, United Kingdom; ^2^National Institute of Mental Health, National Institutes of Health, Bethesda, Maryland 20892; ^3^Computational and Biological Learning Laboratory, Department of Engineering, University of Cambridge, Cambridge CB2 1PZ, United Kingdom; ^4^Adult Eating Disorders Service, Cambridgeshire and Peterborough NHS Foundation Trust, Cambridge CB21 5EF, United Kingdom; ^5^Wellcome-MRC Institute of Metabolic Science, University of Cambridge, Cambridge CB2 0QQ, United Kingdom

## Abstract

Binge eating is a distressing, transdiagnostic eating disorder symptom associated with impulsivity, particularly in negative mood states. Neuroimaging studies of bulimia nervosa (BN) report reduced activity in frontostriatal regions implicated in self-regulatory control, and an influential theory posits that binge eating results from self-regulation failures under stress. However, there is no direct evidence that psychological stress impairs self-regulation in binge-eating disorders, or that any such self-regulatory deficits generalize to binge eating in underweight individuals (i.e., anorexia nervosa bingeing/purging subtype; AN-BP). We therefore determined the effect of acute stress on inhibitory control in 85 women (BN, 33 women; AN-BP, 22 women; 30 control participants). Participants underwent repeated functional MRI scanning during performance of the Stop-signal anticipation task, a validated measure of proactive (i.e., anticipation of stopping) and reactive (outright stopping) inhibition. Neural and behavioral responses to induced stress and a control task were evaluated on 2 consecutive days. Women with BN had reduced proactive inhibition, while prefrontal responses were increased in both AN-BP and BN. Reactive inhibition was neurally and behaviorally intact in both diagnostic groups. Both AN-BP and BN groups showed distinct stress-induced changes in inferior and superior frontal activity during both proactive and reactive inhibition. However, task performance was unaffected by stress. These results offer novel evidence of reduced proactive inhibition in BN, yet inhibitory control deficits did not generalize to AN-BP. Our findings identify intriguing alterations of stress responses and inhibitory function associated with binge eating, but they counsel against stress-induced failures of inhibitory control as a comprehensive explanation for loss-of-control eating.

**SIGNIFICANCE STATEMENT** Binge eating is a common psychiatric syndrome that feels uncontrollable to the sufferer. Theoretically, it has been related to reduced self-regulation under stress, but there remains no direct evidence for this link in binge-eating disorders. Here, we examined how experimentally induced stress affected response inhibition in control participants and women with anorexia nervosa and bulimia nervosa. Participants underwent repeated brain scanning under stressful and neutral conditions. Although patient groups had intact action cancellation, the slowing of motor responses was impaired in bulimia nervosa, even when the likelihood of having to stop increased. Stress altered brain responses for both forms of inhibition in both groups, yet performance remained unimpaired. These findings counsel against a simple model of stress-induced disinhibition as an adequate explanation for binge eating.

## Introduction

Anorexia nervosa (AN) and bulimia nervosa (BN) are eating disorders (EDs) that share cardinal symptoms, including recurrent binge eating and compensatory behaviors (e.g., vomiting). Binge eating occurs in both BN and the binge eating and purging subtype of AN (AN-BP; American Psychiatric Association, 2013a), and it engenders substantial distress and impairment (Udo and Grilo, 2018). Although binge eating has been related to aberrant reward and self-regulatory processing (Schienle et al., 2009; Frank et al., 2011; Berner and Marsh, 2014), its pathophysiological correlates remain poorly characterized.

An influential model posits that binge eating emerges following negative affective states, which reduce an individual's capacity for self-control, thereby leading to loss-of-control eating (Heatherton and Baumeister, 1991). While elevated trait impulsivity in BN (Fischer et al., 2008) and AN-BP (Hoffman et al., 2012) lends support to this model, experimental studies of self-regulation are more equivocal because of inconsistencies across neural and behavioral findings (Lock et al., 2011). For example, fMRI studies of adolescent (Marsh et al., 2011) and adult (Marsh et al., 2009; Skunde et al., 2016) BN report reduced frontostriatal activity during conflict and action inhibition trials on Simon Spatial and Go/NoGo tasks, respectively, yet behavioral impairments were observed only on the Simon Spatial task in adult patients with BN. Altered brain activity without behavioral impairment could indicate either inefficient or compensatory neural responses to preserve task performance. Interestingly, despite unaffected Stop-signal performance, augmented medial prefrontal and anterior cingulate cortex (ACC) activity on failed Stop-signal trials has predicted the subsequent onset of ED behaviors (Bartholdy et al., 2019).

Inconsistencies across levels of analysis and cognitive tasks could partly reflect heterogeneity within the theoretical construct of “self-control.” Behavioral and neurobiological data support related but dissociable forms of impulsivity, including temporal impulsivity and response inhibition, or “inhibitory control,” which is the capacity to slow or stop a response tendency (Dalley et al., 2011). As binge-eating episodes are characterized by a sense that one cannot stop eating (i.e., an ongoing behavior), inhibitory control tasks perhaps best model this behavior. Theoretical frameworks suggest that inhibitory control is modulated by proactive (i.e., goal-directed preparation of stopping) and reactive (stimulus-driven action cancellation) processes (Aron, 2011), which have both shared and unique neural correlates. Bilateral frontoparietal and basal ganglia regions form a broad inhibitory control network that subserves both processes, but bilateral superior parietal and right-dominant, frontal, temporal, and parietal regions have been uniquely related to proactive and reactive inhibition, respectively (Zandbelt et al., 2013; van Belle et al., 2014). Therefore, distinctions between proactive and reactive inhibition should be considered when interrogating self-regulatory impairments associated with binge EDs.

Finally, efforts to validate the model must consider the impact of mood states on self-regulatory control. Although momentary stress precedes binge eating and purging in BN (Berg et al., 2013) and AN (Culbert et al., 2016), it is unknown whether inhibitory control mediates this association. Acute stress increases palatable food preference among male dieters, which co-occurs with augmented fronto-limbic-striatal functional connectivity and reduced connectivity between the ventromedial and dorsolateral prefrontal cortex (dlPFC; Maier et al., 2015). Thus, stress may impair goal-directed prefrontal control, instead evoking habitual responding to food. Indeed, stress-induced decreases in bilateral precuneus, ACC, and dlPFC responses to palatable food cues in BN moderated the association between stress and binge eating in daily life (Fischer et al., 2017).

Here, we investigated the effect of acute stress on two key inhibitory modes in women with AN-BP and BN, and in unaffected control participants. Participants attended a 2 d inpatient study session, which included repeated fMRI scanning under neutral and stressful conditions. Patient groups were expected to have reduced reactive inhibition and inferior frontostriatal activity at baseline, which would be exacerbated by acute stress. We predicted baseline proactive inhibition to be reduced in BN but augmented in AN-BP compared with control participants, aligning with restrictive AN (AN-R; Bartholdy et al., 2017). However, both groups were expected to show stress-induced proactive inhibition impairments and correspondingly altered frontoparietal activity. Finally, exploratory analyses related inhibitory control measures to laboratory-based eating behavior.

## Materials and Methods

### 

#### Participants

We recruited 85 women (mean ± SD age, 23.96 ± 3.98 years) through posted advertisements, the Beat Eating Disorders charity, and an adult ED service in Cambridgeshire. Eligible volunteers were age 18–40 years, English speaking, had normal or corrected-to-normal vision, and, for patient groups, met DSM*-*5 (*Diagnostic and Statistical Manual of Mental Disorders*, fifth edition) diagnostic criteria for either AN-BP or BN. Healthy control subjects with a lifetime psychiatric disorder were ineligible. Patient volunteers with binge-eating disorder, neurodevelopmental disorders, lifetime serious mental illness (e.g., bipolar or psychotic disorders), and/or substance or alcohol use disorders (SUDs) in the past 6 months were excluded. For all groups, exclusion criteria included the following: left handedness; estimated IQ < 80; body mass index (BMI) > 29.9 kg/m^2^; MRI contraindications (e.g., pregnancy, some metallic implants); metabolic, neurological, or cardiovascular diseases (e.g., anemia); lactation, bariatric surgery; and high nicotine dependence, as per the Fagerström Test for Nicotine Dependence (FTND; Heatherton et al., 1991). While not an exclusion criterion for the study, all participants who were prescribed psychotropic medication reported taking a stable dose for at least 2 weeks before participation, aligning with the recommendations of Frank et al. (2018). The study was approved by the Cambridge East Research Ethics Committee (reference 17/EE/0304), and all participants provided signed informed consent.

Participants were matched on age, IQ and, for BN and HC groups, BMI (*t*_(61)_ = 0.19, *p* = 0.85; Table 1). Moreover, rates of binge eating and purging, current treatment, comorbid psychopathology, and medication use (Extended Data Table 1-1) did not differ significantly between patient groups. All AN-BP participants reported recurrent objective binge eating, and the majority (*n* = 19) endorsed purging behaviors.

**Table 1.** Clinical and demographic information

Characteristics	AN (*n* = 22)		BN (*n* = 33)		HC (*n* = 30)		Analysis	
	Mean	SD	Mean	SD	Mean	SD	Χ*^2^*(df), *F*_(df)_, W, *t*_(df)_	*p* Value
Age (years)	24.6	4.7	23.6	3.9	23.9	3.5	Χ*^2^*(2) = 0.8	0.69
BMI (kg/m^2^)	16.4	1.4	22.0	2.4	21.9	2.1	Χ*^2^*(2) = 48.4	<0.001
IQ^*a*^	116	5	114	5	114	5	Χ*^2^*(2) = 3.2	0.21
Age of onset (years)	15.6	2.4	16.2	3.1			*t*_(51.8)_ = −0.8	0.42
Illness duration (years)	9.0	5.8	7.4	4.0			*t*_(34.4)_ = 1.1	0.27
Beck Depression Inventory	35.3	12.0	32.7	10.5	2.4	2.8	Χ*^2^*(2) = 57.7	<0.001
Trait Anxiety Inventory	63.1	10.4	62.8	7.3	33.0	6.9	*F*_(2)_ = 151.1	<0.001
Barratt Impulsiveness Scale	66.2	14.0	68.4	11.1	56.7	6.3	*F*_(2)_ = 10.4	<0.001
Eating Disorder Examination Questionnaire	4.4	0.8	4.6	0.8	0.2	0.2	Χ*^2^*(2) = 58.0	<0.001
Eating disorder examination ratings*^b^*								
Objective binge-eating episodes	38.1	47.9	23.0	29.1			W = 317.5	0.43
Subjective binge-eating episodes	9.5	12.8	6.6	6.2			W = 341.5	0.93
Vomiting episodes	43.5	51.6	24.2	31.0			W = 304.0	0.31
Laxative episodes	1.1	3.4	2.0	3.9			W = 421.5	0.18
Exercise episodes	7.4	13.6	10.9	9.4			W = 478.5	0.04
	*N*	%	*N*	%	*N*	%	Χ*^2^*(df)	*p* Value
Comorbid diagnoses								
Anxiety disorder	3	13.6	3	9.1			Χ*^2^*(1) = 0.3	0.69
Major depressive episode	15	68.2	16	48.5			Χ*^2^*(1) = 2.1	0.15
Personality disorder	2	9.1	5	15.2			Χ*^2^*(1) = 0.4	0.69
Any current treatment	13	59.0	15	45.5			Χ*^2^*(1) = 1.0	0.32
Psychotherapy	9	40.9	9	27.3			Χ*^2^*(1) = 1.1	0.29
Medication	10	45.5	10	30.3			Χ*^2^*(1) = 1.3	0.25
Prior restrictive AN	14	63.6	10	30.3			Χ*^2^*(1) = 6.0	0.01

Group differences were evaluated using one-way ANOVA and, for non-normally distributed data, the nonparametric Kruskal–Wallis test. The two-samples *t* test (two-sided), Mann–Whitney *U* test, and χ^2^ test were used to assess differences between AN and BN groups. For type and dose of prescribed medications, see Extended data Table 1-1.

*^a^*Estimated full-scale IQ from the National Adult Reading Test.

*^b^*ED examination ratings reflect counts over the previous 28 d.

10.1523/JNEUROSCI.2853-20.2021.t1-1 Table 1-1 Supplementary Table 1-1. Download Table 1-1, DOCX file.

#### Study design

**Figure 1.**
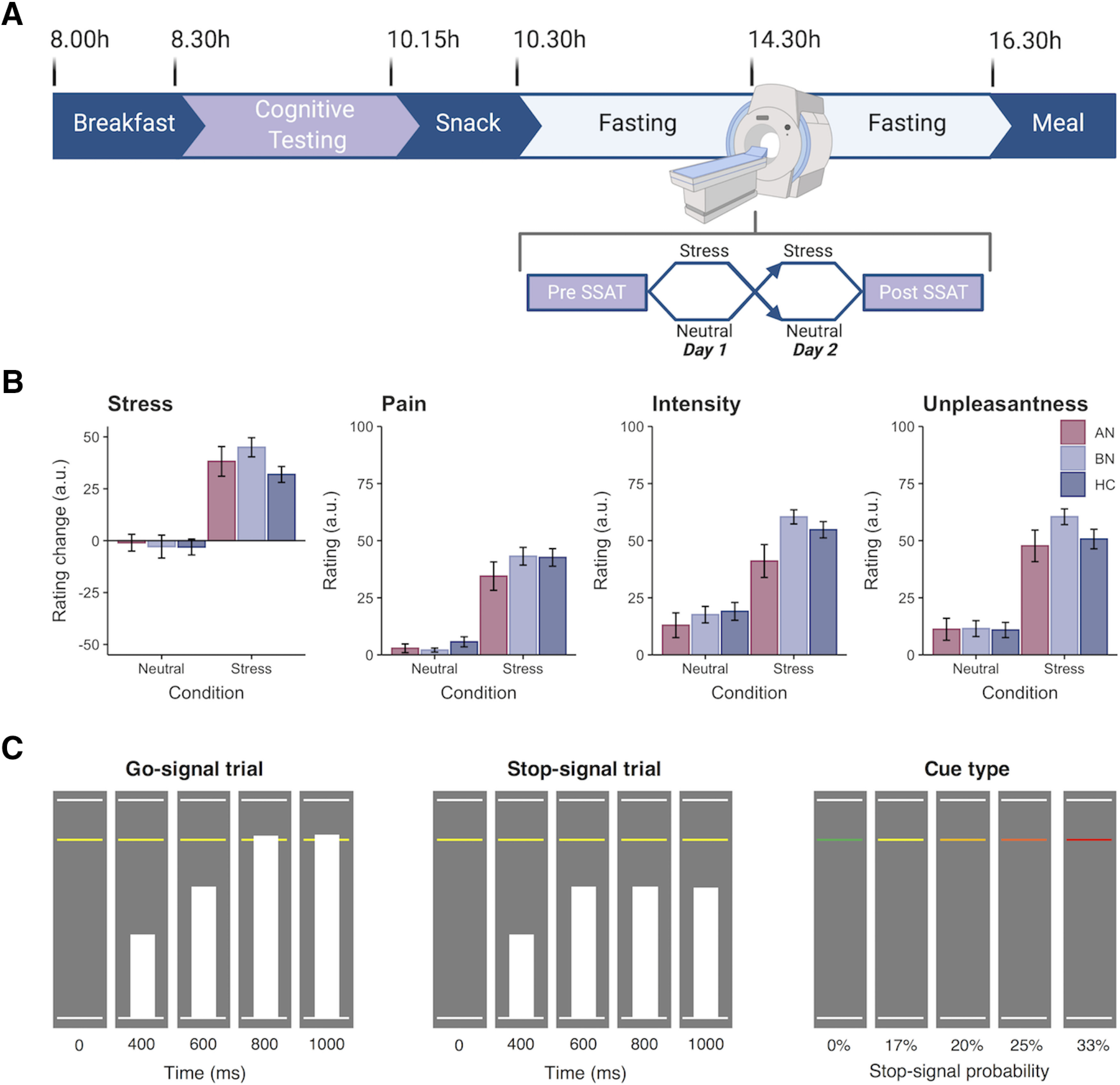
Overview of study design and Stop-signal anticipation task. ***A***, Diagram of inpatient study protocol with representative timeline. Participants were randomized to either a stress induction or control task on each day, which was completed in the MR scanner. Created with BioRender.com. See Extended Data Figure 1-1 for plasma cortisol responses (percentage change from baseline) to the stress and control tasks. ***B***, Participant ratings of subjective stress and electrical stimulation. The stress manipulation induced a significantly greater change in subjective stress compared with the neutral task. Participants rated the electrical stimulation as more painful, intense, and unpleasant following stress as compared with the control task, where stimulation was intended to be detectable but not unpleasant (Westwater et al., 2020). Ratings did not differ significantly by group (all *p* values >0.05). Error bars indicate the SEM. ***C***, Schematic of SSAT trial types adapted from Zandbelt and Vink (2010). Left, On Go-signal trials, participants were instructed to respond when a moving bar reached the middle line. The target response time was 800 ms on each 1000 ms trial (1000 ms intertrial interval). Middle, A minority of trials (25%) were Stop-signal trials, where the moving bar stopped automatically before reaching the middle line. Participants were instructed to withhold their response in the event of a Stop-signal. Right, To index proactive inhibition, the probability of a Stop-signal occurring on a given trial ranged from 0% to 33%, as indicated by colored cues. Participants were told that Stop-signals would never occur on green (baseline) trials, but the likelihood of a Stop-signal occurring increased across yellow to red trials.

Participants underwent the same study procedure, as described previously (Westwater et al., 2020; Fig. 1*A*). Briefly, potential volunteers completed a telephone screening and self-report questionnaire of psychopathology symptoms (American Psychiatric Association, 2013b) before attending an outpatient screening session at Addenbrooke's Hospital (Cambridge, UK). One hundred eligible volunteers completed the outpatient screening session, where they provided informed consent and a fasting blood sample for the assessment of full blood count and thyroid hormones. Then, participants' height, weight, and body composition (via dual-energy X-ray absorptiometry) were measured before a clinical assessment, in which the Eating Disorder Examination (version 16; (Cooper and Fairburn, 1987) and Structured Clinical Interview for DSM*-*5 (First et al., 2015) were administered to determine ED diagnoses and comorbid psychopathology, respectively. Participants also completed the National Adult Reading Test (Blair and Spreen, 1989) to determine their estimated IQ, and the FTND was used to assess nicotine dependence. To reduce participant burden, patient participants who lived outside of Cambridgeshire (*n* = 12) completed the screening session remotely. Participants who underwent remote screening completed all blood sampling and anthropometric measurements during the overnight study session.

Eighty-five women (AN-BP, *n* = 22 women; BN, *n* = 33 women; HC, *n* = 30 women) were eligible for the 2 d overnight study session. Study sessions began at either 8:00 A.M. or 9:00 A.M., and participants' height and weight were measured before a standardized breakfast and a cognitive testing battery. Following a mid-morning snack, participants began a 6 h fast. A cannula was placed ∼1 h before MRI scanning on day 1, and blood samples for cortisol and gut hormones were acquired at fixed timepoints (Westwater et al., 2020). Participants began MRI scanning between 1:30 P.M. and 2:30 P.M. to control for diurnal fluctuations in cortisol. While scanning, participants performed the Stop-signal anticipation task (SSAT; Zandbelt and Vink, 2010) twice, immediately premanipulation and postmanipulation, and manipulation order (stress vs neutral) was counterbalanced across participants. Then, participants had an unsupervised *ad libitum* meal, and those who did not meet their estimated energy requirements were offered an evening snack. This free-choice meal simulated naturalistic circumstances under which participants who suffer with binge eating would experience urges to binge, where stress-induced increases in consumption would lend support to theoretical models of binge eating (Heatherton and Baumeister, 1991). The study protocol was identical on day 2, and participants were discharged following the meal.

#### Stop-signal anticipation task

The SSAT measures both proactive and reactive inhibition. “Proactive inhibition” describes a goal-directed process, elicited by predictive cues, which restrains actions in preparation for stopping. In contrast, “reactive inhibition” is a stimulus-driven process, where a salient signal triggers action cancellation. Task stimuli were presented using Presentation software (version 20; Neurobehavioral Systems), and code may be retrieved from https://github.com/bramzandbelt/SSAT.

As described previously (Zandbelt and Vink, 2010) and in Figure 1*C*, a background of three horizontal lines was present throughout the task. On each trial, a bar moved at a constant speed from the bottom line, reaching the top line in 1000 ms. The main task (i.e., Go-signal trials) involved stopping the moving bar as it reached the middle line by pressing a button with one's right index finger, yielding a target response time (RT) of 800 ms. On a minority of trials, Stop-signals were presented where the moving bar stopped automatically before reaching the middle line. Participants were instructed to withhold their response in the event of a Stop-signal. The probability of a Stop-signal occurring on a given trial ranged from 0% to 33% and was indicated by the color of the middle line (green, 0%; yellow, 17%; amber, 20%; orange, 25%; red, 33%).

The initial Stop-signal onset time was set to 500 ms (i.e., 300 ms before the target response time) for each Stop-signal probability level. Throughout the task, the Stop-signal onset time was adjusted using a staircase procedure (with steps of 25 ms) depending on stopping accuracy, ensuring approximately equal numbers of successful and failed Stop-signal trials.

Trials were presented in either baseline or experimental “blocks” that were composed of 12–15 trials each. The interstimulus interval was 1000 ms. During baseline blocks, participants responded to trials in which the Stop-signal probability was 0%, as indicated by the green Stop-signal probability cue. Experimental blocks were composed of Go-signal trials with a Stop-signal probability >0% (i.e., nongreen cues) and Stop-signal trials (also nongreen cues). Stop-signal trials occurred pseudorandomly throughout experimental blocks, and the Stop-signal probability level varied across trials. Distinct trial orders were used for preinduction and postinduction runs to account for practice effects within each day, where the trial orders were the same across participants and scan sessions. Simulations to determine the optimal trial order indicated that correlations between the different model regressors were sufficiently weak to generate parameter estimates.

In total, the SSAT included 474 trials: 234 Go-signal trials with a Stop-signal probability of 0%, 180 Go-signal trials with a Stop-signal probability >0% (30 yellow, 48 amber, 54 orange, 48 red), and 60 Stop-signal trials (6 yellow, 12 amber, 18 orange, 24 red). In other words, the proportion of Stop-signal trials was 25%. Two 24 s rest blocks were presented after one-third and two-thirds of the trials had elapsed. The task duration was 16 min 36 s. Participants completed a behavioral practice session before fMRI scanning on day 1, in which they were trained on the Go and Stop tasks. Participants were notified that it was equally important to stop the moving bar at the target and to withhold their response in the presence of a Stop-signal. We informed participants that Stop-signals would never occur on trials with green cues, and the likelihood of a Stop-signal occurring was lowest on “yellow” cue trials and highest on “red” cue trials, increasing as the cue color transitioned to red. On day 2, participants were reminded of the task instructions before scanning.

#### Stress induction

To enable within-subject assessment of stress responses, participants completed either an acute, psychological stress induction or a control task (i.e., neutral condition) on each day. In each condition, participants solved multiple-choice, mental math problems of varying difficulty while in the MR scanner; however, participants were motivated to respond accurately in the stress induction, whereas performance was not evaluated during the control task. Moreover, incorrect responses elicited negative feedback (e.g., “Your performance is below average.”) in the stress task, and uncontrollability, a central aspect of psychological stress, was engendered through the delivery of mild electrical stimulation to the abdomen at variable frequencies and intensities. Importantly, subjective ratings of stimulation intensity, unpleasantness, and pain did not differ significantly across groups, indicating that abdominal stimulation was suitable for ED participants (Fig. 1*B*). Subjective stress ratings were collected immediately preinduction and postinduction, and these served to validate the stress manipulation. As psychological stress is inherently grounded in one's subjective experience of the stressor, self-report ratings were viewed as the primary index of stress rather than physiological correlates, which vary substantially across sexes (e.g., cortisol) and remain poorly characterized in women (Kajantie and Phillips, 2006; Ali et al., 2020). Details of the task structure have been described previously (Westwater et al., 2020) and are summarized in the following sections.

##### Electrical stimulation.

Throughout each task, “physical distractors” were delivered to the abdomen in the form of mild electrical stimulation, using a DS7A constant current stimulator (Digitimer). Before MRI scanning, the intensity of electrical stimulation was calibrated for each participant to account for interindividual variability in shock tolerance. Two radio-translucent electrodes (model EL509, BIOPAC Systems) were filled with isotonic paste (model GEL101, BIOPAC Systems) and positioned to the right of the subject's navel, between dermatomes T10 and T12. During the calibration procedure, participants indicated (1) when the stimulation was detectable but not uncomfortable, corresponding to pain ratings of 0–2; and (2) when the stimulation first became uncomfortable but not painful (pain ratings of 5–7; 0, no pain; 10, very painful). Each shock pulse lasted 500 μs.

For the stress induction, shocks were delivered in 5–20 pulse sequences with an interpulse interval range of 0.1–1 s and an intertrain interval range of 0.1–3.9 s, which were randomly sampled in MATLAB (version 2017b; MathWorks). Shock intensity was manually adjusted between the participant's two threshold values throughout the induction. For the control task, stimulation was delivered at predictable intervals and a constant intensity, corresponding to the participant's detection threshold. Trains consisting of five pulses were delivered at an interpulse interval of 0.55 s with an intertrain interval of 2 s. Shock delivery was not contingent on performance. We instructed participants to verbally communicate if the stimulation became painful at any time, in which case it would be reduced. No participants reported discomfort during the tasks, and subjective ratings of the stimulation were acquired immediately following the task.

##### Mental arithmetic control and stress task.

Math task stimuli were presented in MATLAB, using Psychophysics Toolbox (version 3; Brainard, 1997), and code is available at https://github.com/mwestwater/STRIvE-ED. On each day, participants completed 25 practice problems of variable difficulty, and they were instructed to try their best to select the correct answer without taking too much time. Stimuli were presented for a maximum of 30 s, and participants had to respond by selecting one of the three choices. Feedback was presented for 2500 ms either 500 ms after the response or after the 30 s period elapsed. The next trial was presented following a variable interval (500–2500 ms; jitter, 100 ms).

Both the stress induction and control task included 48 multiple-choice mental arithmetic problems, which were matched on difficulty. Before the stress induction, participants were encouraged to respond accurately, and they were informed that only data from participants whose performance met the average group accuracy could be used in the study. Additionally, they were told that physical distractors would be delivered to their abdomen, and that they would be monitored on a live video feed to check that they were paying attention to the task. Conversely, before the control task, participants were told that their performance would not be evaluated and that they would not be watched. Both tasks had the same trial structure as the practice task; however, for the stress induction, the initial stimulus presentation and response time (30 s in the practice task) was set to 10% less than the participant's average response time on the practice task. Accurate responses on three consecutive trials shortened the maximal response window by 10% to ensure low performance. As the sliding response window reduced the overall task duration, the intertrial interval was set to 6 s on every sixth trial to ensure that the task was sufficiently long for the stress induction to be effective. Participants received negative feedback to nonresponses and incorrect responses, whereas no feedback was provided for correct responses. At the end of the task, participants were informed that their performance did not meet the group average. For the control task, the stimulus presentation and response time was 30 s on each trial, and feedback was only provided to indicate correct responses.

#### Image acquisition

MR scanning was completed at the Wolfson Brain Imaging Center at Addenbrooke's Hospital on a 3 T Siemens Skyra^Fit^ scanner, fitted with a 32-channel, GRAPPA (generalized autocalibrating partial parallel acquisition) parallel-imaging head coil. On each day, 1.0 mm isotropic T1-weighted structural images were acquired (TE = 2.95 ms; TR = 2300 ms; flip angle = 9°; acquisition matrix = 256 × 256 mm). Echoplanar images were acquired across 30 interleaved slices with the following parameters: TR = 1600 ms; TE = 23 ms; flip angle = 78°; acquisition matrix = 64 × 64; 3.0 mm isotropic voxels; 631 volumes. One participant was excluded for an incidental finding of white matter abnormalities, and this participant was followed up clinically.

#### Data analysis—SSAT performance

We assessed proactive inhibition by examining the effect of Stop-signal probability on RT, where participants tend to slow responding as the likelihood of having to stop increases (Vink et al., 2005, 2006; Verbruggen and Logan, 2009a; Zandbelt and Vink, 2010). Impaired proactive inhibition would be evident in a failure to increase RT when Stop-signal probability increases, as this would suggest weaker anticipation of stopping. Reactive inhibition was indexed as Stop-signal reaction time (SSRT), which represents the latency of the inhibition process. SSRT was computed using the integration method (Verbruggen and Logan, 2009b) across all Stop-signal probability levels with go omission replacement (Verbruggen et al., 2019). Slower SSRTs would reflect greater latency of the inhibitory process and therefore impaired reactive inhibition.

Behavioral data were analyzed in R (R Core Team, 2015). Aligning with previous reports (Zandbelt and Vink, 2010; Zandbelt et al., 2011), Go-signal RTs that were >1.5 times the interquartile range below the 25th percentile or above the 75th percentile of the RT distribution at each probability level, as well as on failed Stop-signal trials, were defined as outliers. To minimize positive skew, a rank-based inverse normal transformation was applied to RTs (R package *RNOmni*; McCaw, 2019) before analysis. Analyses of proactive inhibition (trial RT) and reactive inhibition (SSRT) were conducted using the linear mixed-effects modeling (LMM) R package *nlme* (Pinheiro et al., 2016), where fixed effects of group, condition, and time were included in both models, with random intercepts for within-subject variables nested within the subject's random effect. Additionally, fixed and random effects for probability level (linear and quadratic terms) were included in the proactive inhibition LMM. Group differences were tested via nonorthogonal contrasts, comparing each patient group to the control group, and model results are reported accordingly. Normality of the model residuals was determined by visual inspection of quantile–quantile plots.

#### Data analysis—fMRI

Image data were preprocessed and analyzed using FreeSurfer (version 6.0; Dale et al., 1999; Fischl et al., 1999) and AFNI (Analysis of Functional NeuroImages) software (Cox, 1996). For each subject, anatomic scans were coregistered with a linear transformation (AFNI program *3dAllineate*) and averaged across days via *3dMean*. The averaged structural image was then processed with the standard FreeSurfer recon-all pipeline. The resulting white matter and ventricle segmentations were resampled to 3 mm isotropic resolution and eroded by 1 voxel along each axis. The remaining preprocessing steps were completed with the afni_proc.py python script, in which functional images were slice time corrected, realigned to the minimum outlier functional volume, coregistered to the subject's skull-stripped averaged anatomical image, nonlinearly warped to the MNI152_T1_2009c template, and smoothed using a 6 mm FWHM kernel. The first three principal components from the time series of lateral third and fourth ventricle sources were estimated and regressed from functional volumes, along with six head motion parameters and their first-order derivatives. Local white matter was regressed from functional volumes using the fast *ANATICOR* pipeline (Jo et al., 2010). Functional volumes with a Euclidean norm motion derivative >0.5 mm were censored, and participants with >10% of volumes censored were excluded from group-level analysis.

Functional MRI data from prestress, poststress, preneutral, and postneutral sessions were available from *n* = 84, *n* = 79, *n* = 80, and *n* = 81 participants, respectively. One participant was excluded from analysis because of white matter abnormalities. In addition, five poststress, four preneutral, and two postneutral runs were excluded because of excessive head motion. A technical error resulted in the exclusion of one additional postneutral session. During a preneutral session, echoplanar image acquisition had to be stopped because of a technical error; however, as ∼70% of functional volumes had been acquired for this participant, their preneutral run was included in the group-level analysis.

Statistical analysis followed a two-level procedure, where successful Stop-signal trials, failed Stop-signal trials, and Go-signal trials with non-0% Stop-signal probability were modeled as regressors of interest in the first-level general linear models. In line with previous work (Zandbelt and Vink, 2010; Zandbelt et al., 2011), we included two amplitude modulators, RT and Stop-signal probability level, for Go-signal trials. AFNI models one regressor for the constant magnitude of the blood oxygenation level-dependent (BOLD) response and separate regressors for each amplitude per timepoint unlike other packages that partition the variance of regressors sequentially. However, as RT (in this context, a measure of the tendency to withhold a response) and Stop-signal probability contrasts may provide complementary information, both were used as measures of proactive inhibition. In addition, incorrect Go-signal trials and rest blocks were included as nuisance regressors; Go-signal trials with a Stop-signal probability of 0% were not modeled, thus constituting an implicit baseline. Regressors were created by convolving γ functions coding for response onset (or Stop-signal delay for successful Stop-signal trials) with a canonical hemodynamic response function. Within each subject run, we computed the following four contrast images: (1) the parametric effect of RT on Go-signal activation (proactive inhibition); (2) the parametric effect of Stop-signal probability on Go-signal activation (proactive inhibition); (3) successful stop versus failed Stop-signal trials (reactive inhibition); and (4) successful Stop-signal versus Go-signal trials with 0% Stop-signal probability (reactive inhibition). We generated two contrasts for reactive inhibition as there is no consensus on which contrast is most appropriate when investigating this inhibitory mode. Beta estimates were determined using restricted maximum likelihood estimation.

**Figure 2.**
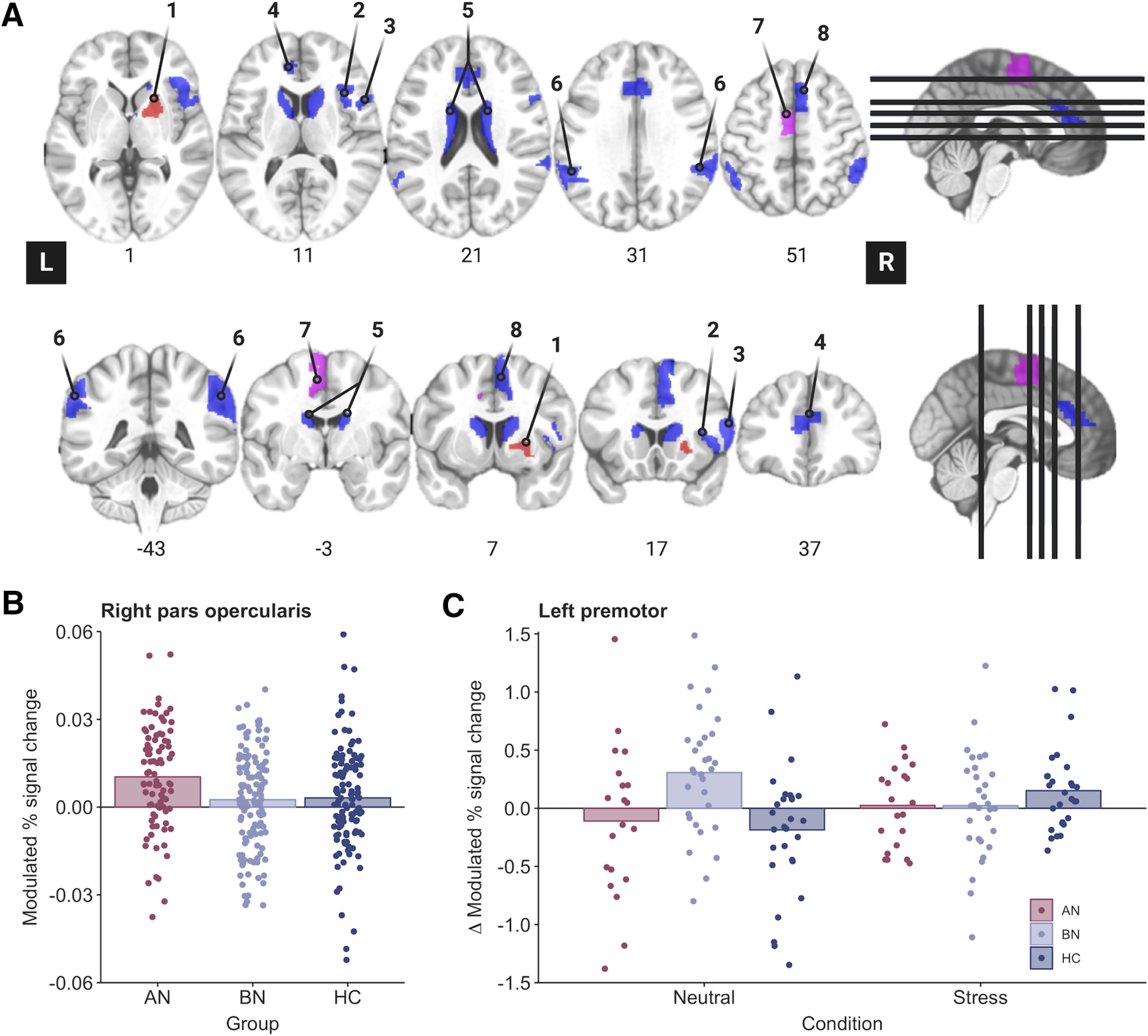
Region of interest analyses identify altered inferior frontal and premotor activity during proactive inhibition in anorexia and bulimia nervosa. ***A***, ROI analyses were conducted in eight regions that have previously been associated with proactive and reactive inhibition (Zandbelt et al., 2011; van Belle et al., 2014), as follows: right putamen (1), right opercular inferior frontal gyrus (2), right ventral inferior frontal gyrus (3), bilateral pregenual anterior cingulate cortex (4), bilateral caudate (5), bilateral superior parietal cortices (6), left premotor cortex (7), and right pre-supplementary motor cortex (8). Blue regions were used in the analysis of both proactive and reactive inhibition, whereas pink and red regions were unique to proactive and reactive analyses, respectively. ROIs are displayed in neurological orientation (L, left). ***B***, The parametric effect of Stop-signal probability was related to increased right inferior frontal gyrus (pars opercularis) activity in the AN-BP group relative to control participants (*p* = 0.005). ***C***, A three-way interaction indicated that the parametric effect of reaction time was related to decreased left premotor activity in the BN group compared with control participants following the stress induction (*p* < 0.001).

We conducted two group analyses for each contrast. First, we examined associations between diagnostic group (AN > HC and BN > HC), condition (stress vs neutral), time (pre vs post), and their interaction and the BOLD response in seven predefined regions of interest (ROIs; Fig. 2*A*). The a priori ROI selection was based on findings from previous functional imaging studies of the SSAT (Zandbelt and Vink, 2010; Zandbelt et al., 2011), proactive and reactive inhibitory control networks (van Belle et al., 2014), and NeuroSynth (https://neurosynth.org) clusters associated with “Stop-signal” and “response inhibition” terms. Averaged β-estimates were extracted from each ROI, as it was defined anatomically in the Brainnetome atlas (Fan et al., 2016), using *3dmaskave*. For each ROI, main and interaction effects were tested in an LMM, and random intercepts for condition and time were included within the random effect of the individual. As seven ROIs were tested per contrast, our α threshold was reduced to *p* = 0.05/7 = 0.007.

Next, we examined whether a group-by-time-by-condition interaction related to differences in whole-brain activation. Whole-brain analyses were completed using the linear mixed-effects modeling AFNI program *3dLME* (Chen et al., 2013), where general linear tests were implemented to test a priori contrasts of interest (e.g., AN > HC, BN > HC, stress > neutral, post > pre). As the model tested a three-way interaction (AN > HC * stress > neutral * post > pre), lower-order interaction and main effects were also included. Both *F*- and *z*-statistics are reported for each effect. The resulting group-level statistical maps were tested for significance using cluster-level inference [cluster-defining threshold, *p* < 0.001, *k* = 18.8; cluster probability, *p* < 0.05, familywise error (FWE) corrected]. Updated versions of *3dFWHMx* and *3dClustSim* were used to correct for multiple comparisons, as these programs incorporate a mixed autocorrelation function to model non-Gaussian noise structure and reduce false-positive rates (Eklund et al., 2016; Cox et al., 2017). For visualization, the mean percentage signal change was extracted from significant whole-brain clusters using *3Dmaskave*.

#### Exploratory analysis of inhibitory control and *ad libitum* consumption

We used LMMs to test whether SSRT, Barratt Impulsiveness scores (BIS-11; Patton et al., 1995), or brain regions implicated in the fMRI analyses explained variance in subsequent food intake (in kilocalories). As described previously (Westwater et al., 2020), one participant declined to initiate the *ad libitum* meal on day 2, and another reported severe nausea before the meal. We therefore modeled observations from 83 participants for these exploratory analyses. For consistency, SSRT and neural responses were modeled from postmanipulation runs only.

Each model included fixed effects of group (AN > HC and BN > HC), condition, and impulsivity measure, where random intercepts for within-subject variables were included within the subject's random effect. Models of SSRT and brain responses also included a person-centered random slope for these variables. Exploratory results were considered statistically significant at *p* = 0.05.

## Results

### Behavioral

#### Manipulation check

As previously reported (Westwater et al., 2020), both subjective stress (Fig. 1*B*) and negative affect were significantly increased following the stress induction relative to the control condition. Moreover, a group-by-condition interaction identified stress-induced plasma cortisol decreases in participants with BN, but not in those with AN-BP, compared with control participants (Extended Data Fig. 1-1; Westwater et al., 2020), aligning with previous reports of blunted stress responses in this disorder (Pirke et al., 1992; Monteleone et al., 2011; Ginty et al., 2012).

10.1523/JNEUROSCI.2853-20.2021.f1-1 Extended Data Figure 1-1 Supplementary Figure 1-1. Download Figure 1-1, DOCX file.

#### Reduced proactive inhibition in bulimia nervosa

We anticipated proactive inhibition would be impaired in BN and augmented in AN-BP while stress-induced impairments would be observed in both groups. RT increased with greater Stop-signal probability (β = 0.01, *t*_(1019)_ = 13.08, *p* < 0.0001); however, this effect was nonlinear, as a significant quadratic probability term suggested that RT slowing plateaued with increasing Stop-signal probability (β = −5.07, *t*_(57,919)_ = −5.28, *p* < 0.0001). RT on non-0% Go-signal trials was significantly decreased postmanipulation (i.e., at time 2; β = −0.14, *t*_(169)_ = −8.49, *p* < 0.0001), which is consistent with the expected practice effects within each scanning session. Moreover, a significant group-by-probability interaction indicated poorer proactive inhibition in the BN group relative to control participants (β = −6.54, *t*_(1012)_ = −2.97, *p* = 0.003; see Fig. 4*A*), where women with BN demonstrated a smaller increase in RT relative to increasing Stop-signal probability. The addition of higher-order interaction terms did not significantly improve model fit (χ^2^(13) = 16.11, *p* = 0.19), indicating that proactive inhibition was not significantly affected by acute stress. RT on 0% Stop-signal probability trials did not differ between AN (*p* = 0.37) or BN (*p* = 0.96) and control participants, indicating equivalent performance on the baseline response task (Table 2).

**Table 2.** SSAT Performance metrics by group and condition

Measure	Group	Neutral		Stress	
		Pre	Post	Pre	Post
		Mean ± 95% CI	Mean ± 95% CI	Mean ± 95% CI	Mean ± 95% CI
SSRT (ms)	AN	273 ± 5	269 ± 7	278 ± 5	268 ± 5
	BN	271 ± 5	268 ± 5	270 ± 5	270 ± 5
	HC	270 ± 6	268 ± 4	271 ± 5	267 ± 4
Go Trial 0%	AN	814.4 ± 1.1	808.7 ± 1.1	818.5 ± 1.2	813.2 ± 1.1
(ms)	BN	820.7 ± 1.0	815.0 ± 0.9	821.0 ± 1.0	816.7 ± 0.9
	HC	823.6 ± 1.1	817.7 ± 1.0	818.9 ± 1.1	814.4 ± 1.0
		Mean (SD)	Mean (SD)	Mean (SD)	Mean (SD)
Stop accuracy	AN	58.7 (4.1)	57.7 (4.7)	58.6 (4.2)	58.8 (3.7)
(%)	BN	60.2 (5.2)	59.3 (6.0)	60.1 (5.2)	58.6 (4.2)
	HC	59.5 (4.7)	59.1 (5.4)	57.5 (5.1)	57.7 (4.7)
Accuracy	AN	98.3 (3.8)	99.1 (0.9)	98.0 (1.9)	99.2 (0.9)
(%)	BN	98.7 (1.3)	98.9 (1.9)	97.4 (5.7)	99.4 (0.7)
	HC	98.0 (3.3)	99.3 (0.9)	98.4 (1.7)	99.3 (0.9)

“Accuracy” represents the percentage of Go-signal trials on which participants made a response.

#### No effect of patient group or stress on reactive inhibition

We predicted that both the AN-BP and BN groups would demonstrate impaired reactive inhibition relative to control participants following the acute stress induction. The significant main effect of time indicated that SSRT was reduced postmanipulation (β = −3.29, *t*_(166)_ = −3.23, *p* = 0.002). However, all other main and interaction effects on SSRT were nonsignificant (all *p* values > 0.05; Table 2). Data met the assumptions of the race model, as evidenced by faster RTs on failed Stop-signal trials compared with Go-signal trials where Stop-signals could occur (β = −21.5, *t*_(339)_ = −39.4, *p* < 0.0001).

### Functional MRI

#### Proactive inhibition

**Figure 3.**
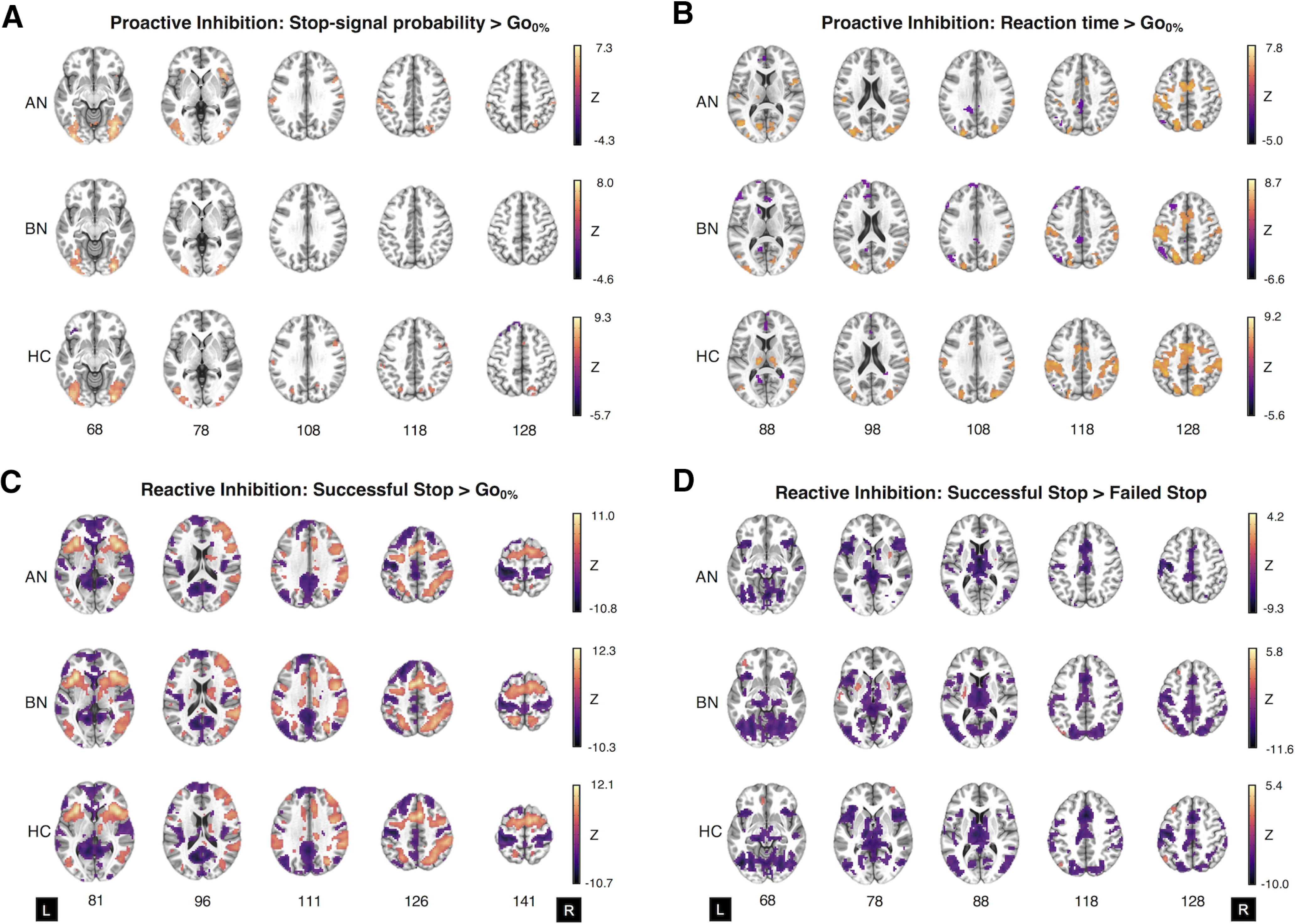
Whole-brain activation in women with anorexia nervosa and bulimia nervosa, and control participants during the Stop-signal anticipation task. ***A–D***, Two-sample *t* tests of the parametric effect of Stop-signal probability versus the implicit baseline (i.e., Go_0%_ trials; ***A***), the parametric effect of reaction time versus the implicit baseline (***B***), successful Stop-signal versus the implicit baseline (***C***), and successful Stop-signal versus failed Stop-signal activation for AN-BP, BN, and control groups (***D***). ***A*** and ***B*** represent proactive inhibition contrasts, whereas ***C*** and ***D*** relate to reactive inhibition. Maps represent significant clusters (voxelwise *p* value < 0.001, FWE cluster probability *p* value < 0.05) and are presented in neurological orientation (L, left). For details on cluster size, coordinates, and associated test statistics, see Extended Data Figures 3-1, 3-2 3-3, 3-4.

Examination of the parametric effects of Stop-signal probability and RT identified increased neural responses across frontoparietal regions that comprise the proactive inhibition network (Fig. 3*A*,*B*, Extended Data Fig. 3-1, Extended Data Fig. 3-2), indicating successful experimental manipulation of proactive inhibition.

##### ROI analyses.

Increasing Stop-signal probability was associated with greater right inferior frontal gyrus (IFG) activity in the AN-BP group relative to control participants (β = 0.007, *t*_(81)_ = 2.91, *p* = 0.005; Fig. 2*B*). IFG activity decreased postmanipulation (i.e., at time 2) across all groups (β = −0.006, *t*_(156)_ = −3.20, *p* = 0.002). In addition, the parametric effect of RT on left premotor cortex activity was related to a three-way interaction, where the BOLD response decreased in participants with BN relative to control participants following the stress induction (β = −0.62, *t*_(151)_ = −3.48, *p* < 0.001; Fig. 2*C*).

##### Whole-brain analyses.

**Figure 4.**
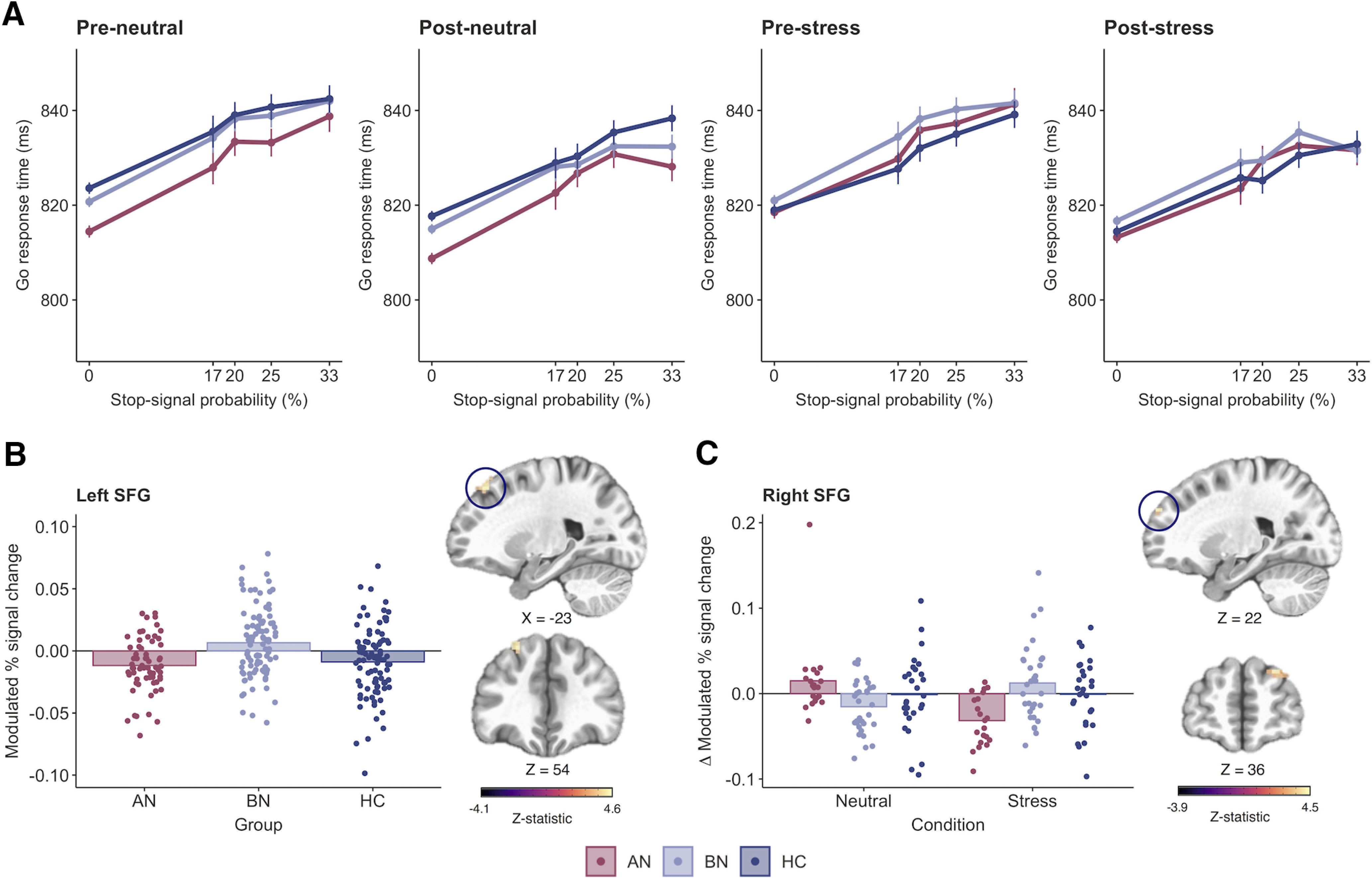
Impaired proactive inhibition in bulimia nervosa is associated with increased superior frontal gyrus activity. ***A***, Reaction time increased as a function of Stop-signal probability in all groups; however, a significant group-by-probability interaction showed that women with BN did not slow to the same degree as control participants in response to increasing Stop-signal probability (*p* = 0.003). ***B***, ***C***, This impairment in proactive inhibition was associated with greater activity in the left superior frontal gyrus (k = 25 voxels, *z* = 4.58, MNI*_X_*_,_*_Y_*_,_*_Z_* = −23, 33, 54; cluster-defining threshold, *p* < 0.001; FWE-corrected cluster probability, *p* < 0.05) in BN relative to control participants. ***C***, A three-way interaction was related to stress-induced increases in the right superior frontal gyrus in women with BN relative to those with AN-BP (k = 34 voxels, *z* = 4.52, MNI*_X_*_,_*_Y_*_,_*_Z_* = 22, 54, 36; cluster-defining threshold, *p* < 0.001; FWE-corrected cluster probability, *p* < 0.05). The size, coordinates, and test statistics of significant clusters from the whole-brain linear mixed-effects analysis of proactive inhibition are reported in Extended Data Figure 4-1. Results are displayed in neurological orientation (L, left). Individual values are overlaid on the mean modulated percentage signal change by group. Error bars indicate the SEM.

Increasing RT was related to reduced left supplementary motor area (SMA) activity postmanipulation (Extended Data Fig. 4-1). Moreover, the effect of Stop-signal probability was significantly affected by time, where activity across the proactive inhibition network generally decreased postmanipulation (Extended Data Fig. 4-1). In line with behavioral findings, the effect of Stop-signal probability also differed significantly by group, where the parameter was related to increased activity in the left superior frontal gyrus (SFG) in BN relative to control participants (k = 25 voxels, *z* = 4.58; Fig. 4*B*, Extended Data Fig. 4-1). A significant three-way interaction was associated with right SFG activity (k = 19 voxels, *F*_(22,31)_ = 10.77). As this effect was not captured by our a priori contrasts, we conducted an additional general linear test, which examined the three-way interaction in BN versus AN-BP. This test indicated augmented SFG activity in BN relative to AN-BP following stress (k = 34 voxels, *z* = 4.52; Fig. 4*C*, Extended Data Fig. 4-1) as patient groups had opposing functional responses to stress in this cluster.

#### Reactive inhibition

Analyses of reactive inhibition (Stop > Go-signal and Stop > FailedStop trials) indicated increased neural responses across the inhibitory control network (Fig. 3*C*, Extended Data Fig. 3-3, Extended Data Fig. 3-4) with markedly similar activation patterns across groups.

10.1523/JNEUROSCI.2853-20.2021.f3-1 Extended Data Figure 3-1 Supplementary Figure 3-1. Download Figure 3-1, DOCX file.

10.1523/JNEUROSCI.2853-20.2021.f3-2 Extended Data Figure 3-2 Supplementary Figure 3-2. Download Figure 3-2, DOCX file.

10.1523/JNEUROSCI.2853-20.2021.f3-3 Extended Data Figure 3-3 Supplementary Figure 3-3. Download Figure 3-3, DOCX file.

10.1523/JNEUROSCI.2853-20.2021.f3-4 Extended Data Figure 3-4 Supplementary Figure 3-4. Download Figure 3-4, DOCX file.

10.1523/JNEUROSCI.2853-20.2021.f4-1 Extended Data Figure 4-1 Supplementary Figure 4-1. Download Figure 4-1, DOCX file.

##### ROI analyses.

The main effect of group and all interaction effects were nonsignificant across all ROIs for both reactive inhibition contrasts. A significant main effect of time was related to right pre-supplementary motor cortex (β = −0.02, *t*_(156)_ = −3.51, *p* < 0.001), ACC (β = −0.01, *t*_(156)_ = −2.79, *p* = 0.006), and bilateral superior parietal cortex activity (β = −0.02, *t*_(156)_ = −4.46, *p* < 0.001) on Stop > Go-signal trials, where activity declined postmanipulation. Moreover, the main effects of condition (β = 0.01, *t*_(82)_ = 2.77, *p* = 0.007) and time (β = 0.01, *t*_(156)_ = 3.14, *p* = 0.002) were associated with ACC activity during Stop > FailedStop trials, where deactivation was less negative on the stress day and postmanipulation (i.e., at time 2). As a time-by-condition interaction term was not significantly related to ACC activity, the observed differences likely reflect BOLD variability across scan days that was not specific to the stress induction.

##### Whole-brain analyses.

**Figure 5.**
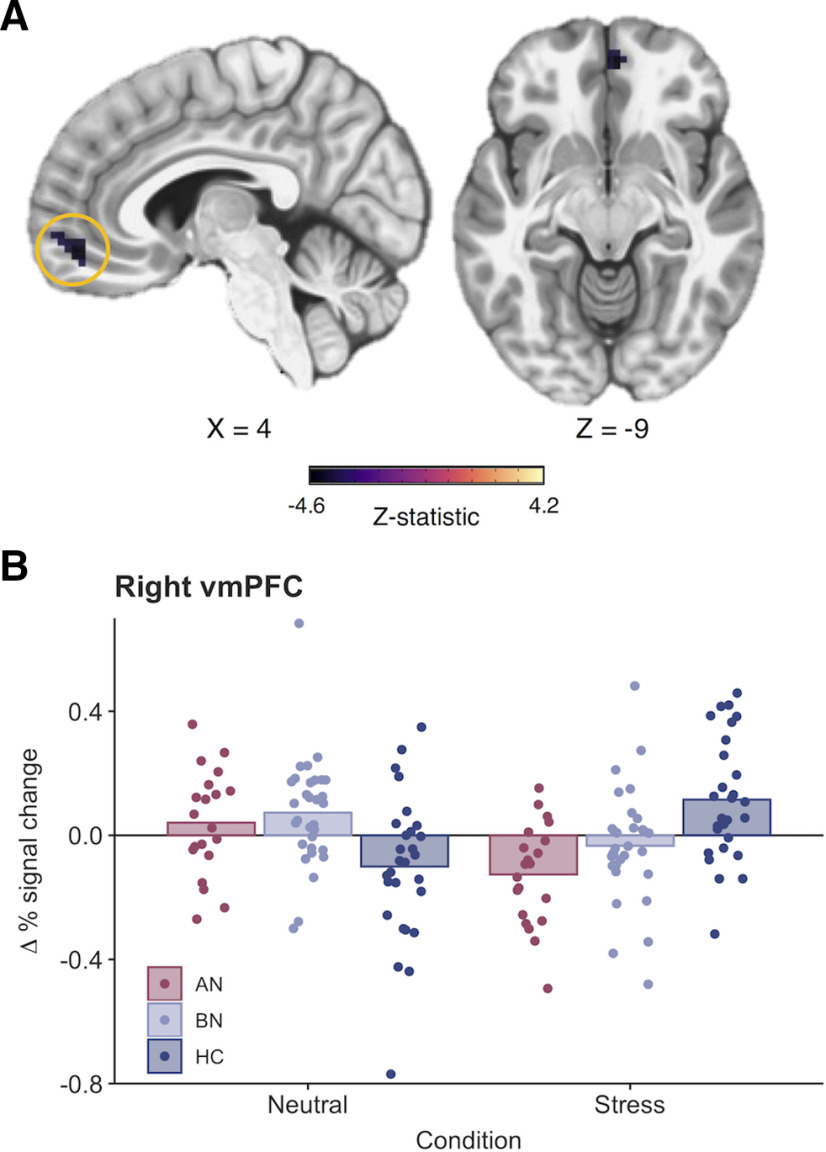
Stress reduces right ventromedial prefrontal cortex activity in women with anorexia nervosa (binge/purge subtype) during reactive inhibition. ***A***, A significant three-way interaction indicated that right vmPFC activity was significantly reduced following acute stress compared with the neutral condition in the AN-BP group relative to the control group (k = 32 voxels, *z* = −4.19, MNI*_X_*_,_*_Y_*_,_*_Z_* = 4, 45, −9; cluster-defining threshold, *p* < 0.001; FWE-corrected cluster probability, *p* < 0.05). The size, coordinates, and test statistics of significant clusters from the whole-brain linear mixed-effects analysis of reactive inhibition are reported in Extended Data Figure 5-1. ***B***, Change in the average percentage signal change for the vmPFC cluster from preinduction to postinduction across conditions. Individual values are overlaid on the mean change in percentage signal change (post – pre) by group.

On Stop > Go-signal trials, neural responses were significantly reduced across the inhibitory control network postmanipulation (Extended Data Fig. 5-1). Activity in left middle temporal, thalamic, posterior insular, occipital, and inferior frontal clusters was reduced postmanipulation during Stop > FailedStop trials. Moreover, left precentral gyrus activity on Stop > FailedStop trials was increased on the stress day relative to the neutral day. Finally, a three-way interaction indicated reduced activity in the right ventromedial PFC (vmPFC) during reactive inhibition (Stop > FailedStop trials) in the AN-BP group relative to the control group following stress (k = 32 voxels, *z* = −4.19; Fig. 5).

10.1523/JNEUROSCI.2853-20.2021.f5-1 Extended Data Figure 5-1 Supplementary Figure 5-1. Download figure 5-1, DOCX file.

#### Associations with food intake

We previously reported that AN-BP and BN groups consumed less in the buffet (Extended Data Fig. 6-1) than the control group, and intake was unaffected by stress (Westwater et al., 2020). On the stress day, women with AN-BP and BN, and control participants consumed [mean (SD)] 898 kcal (872), 873 kcal (409), and 1099 kcal (335), respectively. AN-BP, BN, and control groups ate 849 kcal (806), 941 kcal (560), and 1129 kcal (294), respectively, on the neutral day.

**Figure 6.**
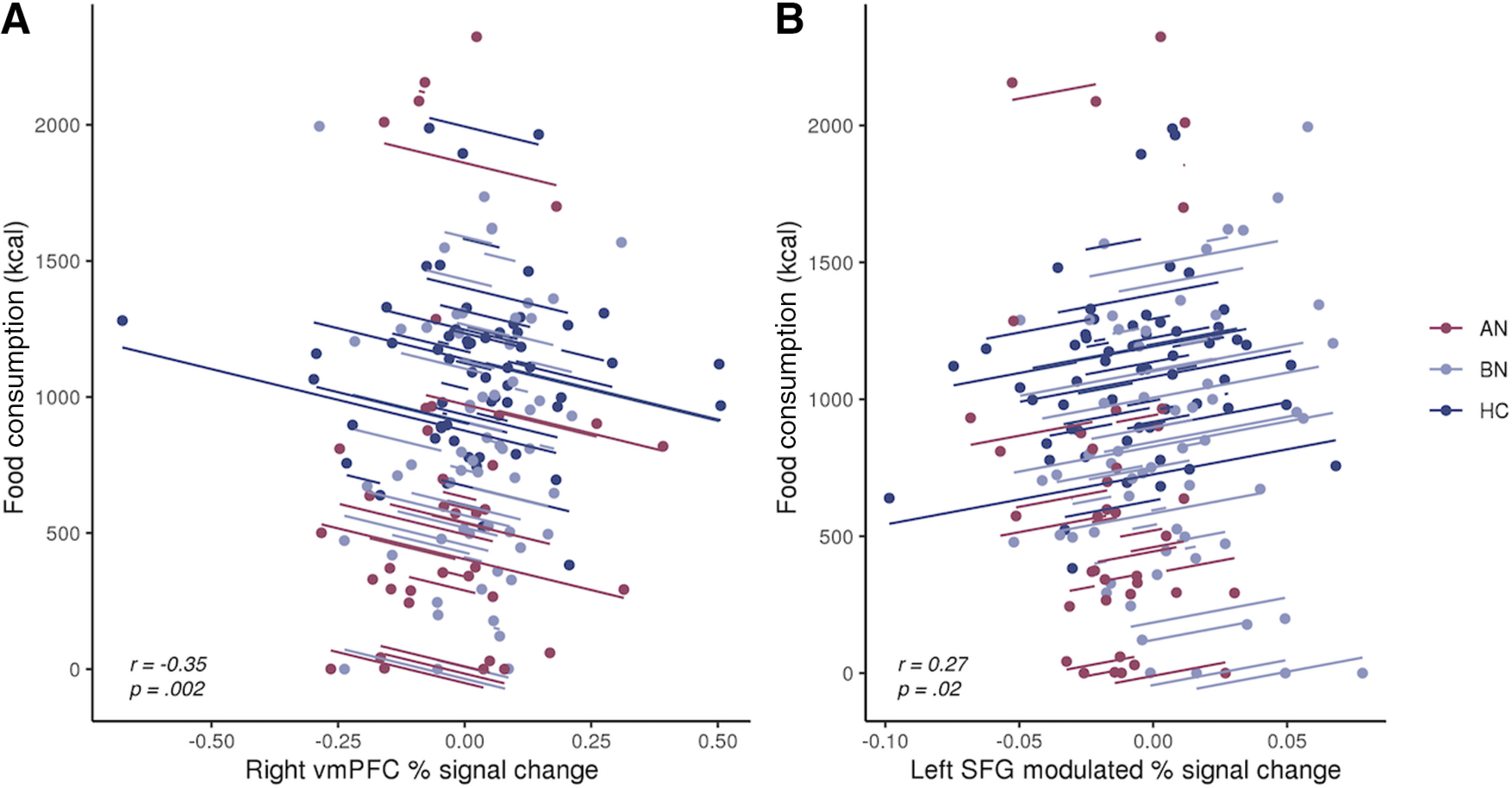
Associations between prefrontal responses during inhibition and *ad libitum* consumption. ***A***, Greater vmPFC responses during reactive inhibition (Successful Stop vs Failed Stop) were negatively related to food consumption during the free-choice meal. For the contents of the meal and corresponding macronutrient information, see Extended Data Figure 6-1. ***B***, Increased left superior frontal gyrus responses to greater Stop-signal probability were positively associated with food intake. Observations within the same participant are modeled with a line of best fit that reflects the overall brain–behavior association. While effects were derived from linear mixed-effects models, repeated-measures correlations were computed for visualization, using the *rmcorr* R package (Bakdash and Marusich, 2017).

Here, we examined whether brain regions demonstrating differing neural responses between groups (e.g., left premotor cortex, right IFG, left SFG) or in a group-by-condition-by-time interaction (right SFG, right vmPFC) explained variance in food intake. Left SFG responses during proactive inhibition predicted increased kilocalorie intake (*z*-scored; β = 4.06, *t*_(71)_ = 2.42, *p* = 0.02), and vmPFC responses during reactive inhibition were negatively related to consumption (β = −1.00, *t*_(71)_ = −3.38, *p* = .0012; Fig. 6). These associations were observed in the full sample and did not differ significantly by group or condition. The effects of SSRT, trait impulsivity, and all interaction terms were nonsignificant (all *p* values > 0.05).

10.1523/JNEUROSCI.2853-20.2021.f6-1 Extended Data Figure 6-1 Supplementary Figure 6-1. Download figure 6-1, DOCX file.

## Discussion

As failed self-regulation in response to stressors has gained traction as a putative mechanism of binge eating, it has become increasingly important to characterize the precise self-regulatory deficits associated with binge-eating disorders. We assessed the impact of induced stress on inhibitory control in women with AN-BP, BN, and matched control participants, reporting three key findings. First, women with BN, but not AN-BP, had reduced proactive inhibition, yet both groups demonstrated increased prefrontal responses during the anticipation of stopping compared with the control group. Second, we found stress-induced changes in the neural correlates of proactive and reactive inhibition, with notable differences across diagnostic groups. Third, AN-BP and BN groups had intact reactive inhibition, and neither proactive nor reactive inhibition performance was affected by acute stress.

We report novel evidence of reduced proactive inhibition in BN relative to control participants, which co-occurred with increased activity in the left dorsolateral SFG. Increased left SFG activity and concurrent performance deficits could reflect inefficient recruitment of other regions within the inhibitory control network, namely inferior and middle frontal gyri, which share reciprocal connections with the SFG (Li et al., 2013). Inefficient or compensatory responses may also explain increased right IFG responses in the AN-BP group during intact proactive inhibition. Alternatively, given the role of the pars opercularis in “braking” motor responses (Swann et al., 2012; Aron et al., 2014), increased activity could reflect improved proactive adjusting in individuals with AN-BP on the neural level, complementing previous behavioral reports in AN-R (Bartholdy et al., 2017). Exploratory analyses found that left SFG responses predicted increased postscan calorie intake. This finding lends additional support to the notion of inefficiencies across the proactive inhibitory network that may relate to abnormal eating behavior, specifically overconsumption. As this association was not moderated by disorder status, this finding might suggest a general–rather than a diagnosis-specific—association between left SFG activation during proactive inhibition and food intake. However, our sample may have lacked sufficient statistical power to detect small interaction effects, and future studies with larger sample sizes will be critical to determining whether and how this relationship differs between AN-BP, BN, and control groups.

Acute psychological stress altered right SFG and left premotor cortex responses during proactive inhibition, as well as right vmPFC activity during outright stopping, differently between groups. Specifically, stress augmented right SFG responses to increasing Stop-signal probability in the BN group relative to the AN-BP group. In women with BN, these stress-induced increases in SFG responses perhaps compensated for concomitant decreases in premotor activity during RT slowing, thus preserving task performance. Indeed, increased prefrontal activity has been reported in healthy adults following pain stress, where activation was presumed to support working memory performance (Porcelli et al., 2008).

One explanation for reduced poststress vmPFC responses in women with AN-BP relative to control participants, who showed augmented responses, could be stress-induced alterations in inter-regional modulation (Veer et al., 2011). The vmPFC is the primary cortical target of limbic projections (Averbeck and Seo, 2008), and stress-induced increases in activity may provide top-down modulation of amygdala reactivity and negative emotions. While not typically associated with inhibitory control, augmented vmPFC activity during reactive inhibition has been reported following methylphenidate administration (Li et al., 2010) and neuromodulation of the pre-SMA (Yu et al., 2015). These findings, together with our observations following acute stress, could implicate norepinephrine signaling in altered vmPFC activation, but further research is needed. Our finding of a negative relationship between vmPFC responses to reactive stopping and postscan calorie consumption suggests that vmPFC activation during inhibition may be important for dietary control. However, as discussed above, the specificity of this brain–behavior association to clinically significant eating pathology remains unclear, and we encourage future replication attempts in larger sample sizes.

Stress-induced reductions in prefrontal responses during both proactive and reactive inhibition in women with AN-BP could reflect the consequences of prolonged extreme stress, namely, significantly low weight, which engenders various cognitive and neuroendocrine perturbations (Delvenne et al., 1995; Misra and Klibanski, 2014). Interestingly, preclinical research has identified disrupted dopaminergic signaling following severe stress (Lemos et al., 2012; Hollon et al., 2015); however, the effect of stress on dopaminergic projections to prefrontal cortex remains understudied. The dearth of research in this area discourages a premature interpretation of our stress induction effects in AN-BP. Instead, findings of task-specific, stress-induced reductions in prefrontal responses in AN-BP participants may inform future investigations into neurocognitive alterations associated with prolonged and increasing stress.

Contrary to our hypotheses, reactive inhibition, indexed as SSRT, was unaffected by diagnostic group or stress, and it was unrelated to free-choice consumption. As we have reviewed, the findings of impaired self-regulatory performance in BN and AN-BP are inconsistent (Marsh et al., 2011; Skunde et al., 2016), and our results suggest that the subjective “loss of control” that characterizes binge-eating episodes does not relate to deficits in one's capacity for action cancellation. While often considered a valid and translational measure of inhibitory control, our findings, and a recent mega-analysis of polysubstance use, question the clinical utility of SSRT. Indeed, the latter found that increased SSRT was not significantly related to various SUDs, including alcohol and cocaine use disorders (Liu et al., 2019). As stress-induced deficits in the ability to delay food reward were found in nonclinical samples (Maier et al., 2015), future research should assess state changes in decision-making as a potential mechanism of loss-of-control eating in clinical groups.

Although our design had notable strengths, several limitations should be considered. First, we recruited a representative sample of women with EDs, and, as expected, the majority suffered with comorbid psychopathology and many used medication. It is difficult to robustly adjust for these in analyses as our modest sample size would render any subgroup analysis of medication- or comorbidity-free participants very underpowered. However, these characteristics may improve the generalizability of our findings as comorbidity and medication use are the norm rather than the exception among individuals with EDs (Fazeli et al., 2012; Udo and Grilo, 2019). Indeed, rates of psychiatric comorbidity (AN-BP participants, 73%; BN participants, 70%) and medication use (AN-BP participants, 46%; BN participants, 30%) in our sample align with those reported in epidemiological studies of EDs (Fazeli et al., 2012; Ulfvebrand et al., 2015). One concern with medication use is a potential impact on response inhibition. Of those individuals using medication, most were prescribed either selective serotonin reuptake inhibitors or serotonin-norepinephrine reuptake inhibitors with high affinity for 5-HT, and 5-HT modulation has been shown to have no effect on response inhibition (Chamberlain et al., 2006). Nevertheless, future studies could attempt to dissociate medication effects by including an unmedicated, positive control group, but such a group would likely differ from the experimental group in other important ways, such as illness severity and treatment duration. Second, despite providing increased statistical power, repeated-measures designs may elicit practice effects. While our design mitigated these effects through counterbalancing, repeated performance of the SSAT on each day could induce within-session training effects. However, preinduction and postinduction task performance militated against the possibility that baseline nonspecific performance differences across the groups could contaminate our results. Moreover, as within-session repetition occurred across both conditions, we are confident that our significant results are specific to induced stress when accounting for potential practice effects. Third, disorder-salient stimuli (e.g., food), which may accentuate or reveal self-regulatory deficits (Wu et al., 2013), were not used, and future study should examine the impact of stress on performance in these contexts. Finally, the conditions under which stress was induced (i.e., in an MR scanner) and eating behavior was assessed differed from those in daily life, and the integration of neuroimaging with prospective, real-world monitoring of internal states and binge-eating behavior would extend our work.

Our findings counsel against a simplistic, stress-induced failure of regulation as an overall explanation for binge eating in individuals diagnosed with AN-BP or BN, underscoring the need for alternative models of these illnesses. Moreover, dissociations across diagnostic groups suggest that models of binge eating based on BN may not apply to AN-BP. Given the complex metabolic and psychological disturbances associated with these disorders, future efforts to identify the neurocognitive mechanisms of binge eating should consider the roles of interacting peripheral physiological processes.

## References

[B1] Ali N, Nitschke JP, Cooperman C, Baldwin MW, Pruessner JC (2020) Systematic manipulations of the biological stress systems result in sex-specific compensatory stress responses and negative mood outcomes. Neuropsychopharmacology 45:1672–1680. 10.1038/s41386-020-0726-8 32498073 PMC7421880

[B2] American Psychiatric Association (2013a) Diagnostic and statistical manual of mental disorders, Ed 5, pp 329–360. Arlington, VA: American Psychiatric Association.

[B3] American Psychiatric Association (2013b) DSM–5 self-rated level 1 cross-cutting symptom measure, adult. In: Diagnostic and statistical manual of mental disorders: DSM-5, Ed 5, pp 734–739. Arlington, VA: American Psychiatric Association.

[B4] Aron AR (2011) From reactive to proactive and selective control: developing a richer model for stopping inappropriate responses. Biol Psychiatry 69:e55–e68. 10.1016/j.biopsych.2010.07.024 20932513 PMC3039712

[B5] Aron AR, Robbins TW, Poldrack RA (2014) Inhibition and the right inferior frontal cortex: one decade on. Trends Cogn Sci 18:177–185. 10.1016/j.tics.2013.12.003 24440116

[B6] Averbeck BB, Seo M (2008) The statistical neuroanatomy of frontal networks in the macaque. PLoS Comput Biol 4:e1000050. 10.1371/journal.pcbi.1000050 18389057 PMC2268011

[B7] Bakdash JZ, Marusich LR (2017) Repeated measures correlation. Front Psychol 8:456. 10.3389/fpsyg.2017.00456 28439244 PMC5383908

[B8] Bartholdy S, Rennalls SJ, Jacques C, Danby H, Campbell IC, Schmidt U, O'Daly OG (2017) Proactive and reactive inhibitory control in eating disorders. Psychiatry Res 255:432–440. 10.1016/j.psychres.2017.06.073 28672226 PMC5555256

[B9] Bartholdy S, O'Daly OG, Campbell IC, Banaschewski T, Barker G, Bokde ALW, Bromberg U, Büchel C, Quinlan EB, Desrivières S, Flor H, Frouin V, Garavan H, Gowland P, Heinz A, Ittermann B, Martinot J-L, Paillère Martinot M-L, Nees F, et al. (2019) Neural correlates of failed inhibitory control as an early marker of disordered eating in adolescents. Biol Psychiatry 85:956–965. 10.1016/j.biopsych.2019.01.027 31122340

[B10] Berg KC, Crosby RD, Cao L, Peterson CB, Engel SG, Mitchell JE, Wonderlich SA (2013) Facets of negative affect prior to and following binge-only, purge-only, and binge/purge events in women with bulimia nervosa. J Abnorm Psychol 122:111–118. 10.1037/a0029703 22985015 PMC3646562

[B11] Berner LA, Marsh R (2014) Frontostriatal circuits and the development of bulimia nervosa. Front Behav Neurosci 8:395. 10.3389/fnbeh.2014.00395 25452718 PMC4233924

[B12] Blair JR, Spreen O (1989) Predicting premorbid IQ: a revision of the national adult reading test. Clinical Neuropsychologist 3:129–136. 10.1080/13854048908403285

[B13] Brainard D (1997) The Psychophysics Toolbox. Spat Vis 10:433–436. 10.1163/156856897X00357 9176952

[B14] Chamberlain SR, Müller U, Blackwell AD, Clark L, Robbins TW, Sahakian BJ (2006) Neurochemical modulation of response inhibition and probabilistic learning in humans. Science 311:861–863. 10.1126/science.1121218 16469930 PMC1867315

[B15] Chen G, Saad ZS, Britton JC, Pine DS, Cox RW (2013) Linear mixed-effects modeling approach to FMRI group analysis. Neuroimage 73:176–190. 10.1016/j.neuroimage.2013.01.047 23376789 PMC3638840

[B16] Cooper Z, Fairburn C (1987) The eating disorder examination: a semi-structured interview for the assessment of the specific psychopathology of eating disorders. Int J Eat Disord 6:1–8. 10.1002/1098-108X(198701)6:1<1::AID-EAT2260060102>3.0.CO;2-9

[B17] Cox RW (1996) AFNI: software for analysis and visualization of functional magnetic resonance neuroimages. Comput Biomed Res 29:162–173. 10.1006/cbmr.1996.0014 8812068

[B18] Cox RW, Chen G, Glen DR, Reynolds RC, Taylor PA (2017) FMRI clustering in AFNI: false-positive rates redux. Brain Connect 7:152–171. 10.1089/brain.2016.0475 28398812 PMC5399747

[B19] Culbert KM, Lavender JM, Crosby RD, Wonderlich SA, Engel SG, Peterson CB, Mitchell JE, Crow SJ, Le Grange D, Cao L, Fischer S (2016) Associations between negative affect and binge/purge behaviors in women with anorexia nervosa: considering the role of negative urgency. Compr Psychiatry 66:104–112. 10.1016/j.comppsych.2016.01.010 26995243 PMC4800336

[B20] Dale AM, Fischl B, Sereno MI (1999) Cortical surface-based analysis. I. Segmentation and surface reconstruction. Neuroimage 9:179–194. 10.1006/nimg.1998.0395 9931268

[B21] Dalley JW, Everitt BJ, and Robbins TW (2011) Impulsivity, compulsivity, and top-down cognitive control. Neuron 69:680–694. 10.1016/j.neuron.2011.01.020 21338879

[B22] Delvenne V, Lotstra F, Goldman S, Biver F, De Maertelaer V, Appelboom-Fondu J, Schoutens A, Bidaut LM, Luxen A, Mendelwicz J (1995) Brain hypometabolism of glucose in anorexia nervosa: a PET scan study. Biol Psychiatry 37:161–169. 10.1016/0006-3223(94)00189-A 7727624

[B23] Eklund A, Nichols TE, Knutsson H (2016) Cluster failure: why fMRI inferences for spatial extent have inflated false-positive rates. Proc Natl Acad Sci U S A 113:7900–7905. 10.1073/pnas.1602413113 27357684 PMC4948312

[B24] Fan L, Li H, Zhuo J, Zhang Y, Wang J, Chen L, Yang Z, Chu C, Xie S, Laird AR, Fox PT, Eickhoff SB, Yu C, Jiang T (2016) The Human Brainnetome Atlas: a new brain atlas based on connectional architecture. Cereb Cortex 26:3508–3526. 10.1093/cercor/bhw157 27230218 PMC4961028

[B25] Fazeli PK, Calder GL, Miller KK, Misra M, Lawson EA, Meenaghan E, Lee H, Herzog D, Klibanski A (2012) Psychotropic medication use in anorexia nervosa between 1997 and 2009. Int J Eat Disord 45:970–976. 10.1002/eat.22037 22733643 PMC3726215

[B26] First MB, Williams JBW, Karg RS, Spitzer RL (2015) Structured clinical interview for DSM-5 disorders—Clinician version (SCID-5-CV). Arlington, VA: American Psychiatric Association.

[B27] Fischer S, Smith GT, Cyders MA (2008) Another look at impulsivity: a meta-analytic review comparing specific dispositions to rash action in their relationship to bulimic symptoms. Clin Psychol Rev 28:1413–1425. 10.1016/j.cpr.2008.09.001 18848741 PMC2677964

[B28] Fischer S, Breithaupt L, Wonderlich J, Westwater ML, Crosby RD, Engel SG, Thompson J, Lavender J, and Wonderlich S (2017) Impact of the neural correlates of stress and cue reactivity on stress related binge eating in the natural environment. J Psychiatr Res 92:15–23. 10.1016/j.jpsychires.2017.03.017 28376408

[B29] Fischl B, Sereno MI, and Dale AM (1999) Cortical surface-based analysis. II: inflation, flattening, and a surface-based coordinate system. Neuroimage 9:195–207. 10.1006/nimg.1998.0396 9931269

[B30] Frank GKW, Reynolds JR, Shott ME, O'Reilly RC (2011) Altered temporal difference learning in bulimia nervosa. Biol Psychiatry 70:728–735. 10.1016/j.biopsych.2011.05.011 21718969 PMC3186835

[B31] Frank GKW, Favaro A, Marsh R, Ehrlich S, and Lawson EA (2018) Toward valid and reliable brain imaging results in eating disorders. Int J Eat Disord 51:250–261. 10.1002/eat.22829 29405338 PMC7449370

[B32] Ginty AT, Phillips AC, Higgs S, Heaney JLJ, Carroll D (2012) Disordered eating behaviour is associated with blunted cortisol and cardiovascular reactions to acute psychological stress. Psychoneuroendocrinology 37:715–724. 10.1016/j.psyneuen.2011.09.004 21962379

[B33] Heatherton TF, Baumeister RF (1991) Binge eating as escape from self-awareness. Psychol Bull 110:86–108. 10.1037/0033-2909.110.1.86 1891520

[B34] Heatherton TF, Kozlowski LT, Frecker RC, Fagerström KO (1991) The Fagerström test for nicotine dependence: a revision of the Fagerström Tolerance Questionnaire. Br J Addict 86:1119–1127. 10.1111/j.1360-0443.1991.tb01879.x 1932883

[B35] Hoffman ER, Gagne DA, Thornton LM, Klump KL, Brandt H, Crawford S, Fichter MM, Halmi KA, Johnson C, Jones I, Kaplan AS, Mitchell JE, Strober M, Treasure J, Woodside DB, Berrettini WH, Kaye WH, Bulik CM (2012) Understanding the association of impulsivity, obsessions, and compulsions with binge eating and purging behaviours in anorexia nervosa. Eur Eat Disord Rev 20:e129–e136. 10.1002/erv.2161 22351620 PMC3443865

[B36] Hollon NG, Burgeno LM, and Phillips PEM (2015) Stress effects on the neural substrates of motivated behavior. Nat Neurosci 18:1405–1412. 10.1038/nn.4114 26404715 PMC4721524

[B37] Jo HJ, Saad ZS, Simmons WK, Milbury LA, and Cox RW (2010) Mapping sources of correlation in resting state FMRI, with artifact detection and removal. Neuroimage 52:571–582. 10.1016/j.neuroimage.2010.04.246 20420926 PMC2897154

[B38] Kajantie E, and Phillips DIW (2006) The effects of sex and hormonal status on the physiological response to acute psychosocial stress. Psychoneuroendocrinology 31:151–178. 10.1016/j.psyneuen.2005.07.002 16139959

[B39] Lemos JC, Wanat MJ, Smith JS, Reyes BAS, Hollon NG, Van Bockstaele EJ, Chavkin C, and Phillips PEM (2012) Severe stress switches CRF action in the nucleus accumbens from appetitive to aversive. Nature 490:402–406. 10.1038/nature11436 22992525 PMC3475726

[B40] Li C-SR, Morgan PT, Matuskey D, Abdelghany O, Luo X, Chang JLK, Rounsaville BJ, Ding Y, Malison RT (2010) Biological markers of the effects of intravenous methylphenidate on improving inhibitory control in cocaine-dependent patients. Proc Natl Acad Sci U S A 107:14455–14459. 10.1073/pnas.1002467107 20660731 PMC2922598

[B41] Li W, Qin W, Liu H, Fan L, Wang J, Jiang T, Yu C (2013) Subregions of the human superior frontal gyrus and their connections. Neuroimage 78:46–58. 10.1016/j.neuroimage.2013.04.011 23587692

[B42] Liu Y, van den Wildenberg WPM, de Graaf Y, Ames SL, Baldacchino A, Bø R, Cadaveira F, Campanella S, Christiansen P, Claus ED, Colzato LS, Filbey FM, Foxe JJ, Garavan H, Hendershot CS, Hester R, Jester JM, Karoly HC, Kräplin A, Kreusch F, et al. (2019) Is (poly-) substance use associated with impaired inhibitory control? A mega-analysis controlling for confounders. Neurosci Biobehav Rev 105:288–304. 10.1016/j.neubiorev.2019.07.006 31319124

[B43] Lock J, Garrett A, Beenhakker J, Reiss AL (2011) Aberrant brain activation during a response inhibition task in adolescent eating disorder subtypes. Am J Psychiatry 168:55–64. 10.1176/appi.ajp.2010.10010056 21123315 PMC3016457

[B44] Maier SU, Makwana AB, Hare TA (2015) Acute stress impairs self-control in goal-directed choice by altering multiple functional connections within the brain's decision circuits. Neuron 87:621–631. 10.1016/j.neuron.2015.07.005 26247866

[B45] Marsh R, Steinglass JE, Gerber AJ, Graziano O'Leary K, Wang Z, Murphy D, Walsh BT, Peterson BS (2009) Deficient activity in the neural systems that mediate self-regulatory control in bulimia nervosa. Arch Gen Psychiatry 66:51–63. 10.1001/archgenpsychiatry.2008.504 19124688 PMC2759684

[B46] Marsh R, Horga G, Wang Z, Wang P, Klahr KW, Berner LA, Walsh BT, and Peterson BS (2011) An fMRI study of self-regulatory control and conflict resolution in adolescents with bulimia nervosa. Am J Psychiatry 168:1210–1220. 10.1176/appi.ajp.2011.11010094 21676991 PMC3328859

[B47] McCaw Z (2019) RNOmni: rank Normal Transformation Omnibus Test (0.7.1). Vienna, Austria: R Foundation.

[B48] Misra M, Klibanski A (2014) Endocrine consequences of anorexia nervosa. Lancet Diabetes Endocrinol 2:581–592. 10.1016/S2213-8587(13)70180-3 24731664 PMC4133106

[B49] Monteleone P, Scognamiglio P, Canestrelli B, Serino I, Monteleone AM, Maj M (2011) Asymmetry of salivary cortisol and α-amylase responses to psychosocial stress in anorexia nervosa but not in bulimia nervosa. Psychol Med 41:1963–1969. 10.1017/S0033291711000092 21284914

[B50] Patton JH, Stanford MS, Barratt ES (1995) Factor structure of the Barratt impulsiveness scale. J Clin Psychol 51:768–774. 10.1002/1097-4679(199511)51:6<768::AID-JCLP2270510607>3.0.CO;2-1 8778124

[B51] Pinheiro J, Bates D, DebRoy S, Sarkar D (2016) nlme: linear and nonlinear mixed effects models. R package version 3.1-128. Vienna, Austria: R Foundation.

[B52] Pirke KM, Platte P, Laessle R, Seidl M, and Fichter MM (1992) The effect of a mental challenge test of plasma norepinephrine and cortisol in bulimia nervosa and in controls. Biol Psychiatry 32:202–206. 10.1016/0006-3223(92)90026-V 1420635

[B53] Porcelli AJ, Cruz D, Wenberg K, Patterson MD, Biswal BB, Rypma B (2008) The effects of acute stress on human prefrontal working memory systems. Physiol Behav 95:282–289. 10.1016/j.physbeh.2008.04.027 18692209

[B54] R Core Team (2015) R: a language and environment for statistical computing. Vienna, Austria: R Foundation.

[B55] Schienle A, Schäfer A, Hermann A, Vaitl D (2009) Binge-eating disorder: reward sensitivity and brain activation to images of food. Biol Psychiatry 65:654–661. 10.1016/j.biopsych.2008.09.028 18996508

[B56] Skunde M, Walther S, Simon JJ, Wu M, Bendszus M, Herzog W, Friederich H-C (2016) Neural signature of behavioural inhibition in women with bulimia nervosa. J Psychiatry Neurosci 41:E69–E78. 10.1503/jpn.150335 27575858 PMC5008924

[B57] Swann NC, Cai W, Conner CR, Pieters TA, Claffey MP, George JS, Aron AR, Tandon N (2012) Roles for the pre-supplementary motor area and the right inferior frontal gyrus in stopping action: electrophysiological responses and functional and structural connectivity. Neuroimage 59:2860–2870. 10.1016/j.neuroimage.2011.09.049 21979383 PMC3322194

[B58] Udo T, Grilo CM (2018) Prevalence and correlates of DSM-5–defined eating disorders in a nationally representative sample of U.S. adults. Biol Psychiatry 84:345–354. 10.1016/j.biopsych.2018.03.014 29859631 PMC6097933

[B59] Udo T, Grilo CM (2019) Psychiatric and medical correlates of DSM-5 eating disorders in a nationally representative sample of adults in the United States. Int J Eat Disord 52:42–50. 10.1002/eat.23004 30756422

[B60] Ulfvebrand S, Birgegård A, Norring C, Högdahl L, von Hausswolff-Juhlin Y (2015) Psychiatric comorbidity in women and men with eating disorders results from a large clinical database. Psychiatry Res 230:294–299. 10.1016/j.psychres.2015.09.008 26416590

[B61] van Belle J, Vink M, Durston S, Zandbelt BB (2014) Common and unique neural networks for proactive and reactive response inhibition revealed by independent component analysis of functional MRI data. NeuroImage 103:65–74. 10.1016/j.neuroimage.2014.09.014 25224995

[B62] Veer IM, Oei NYL, Spinhoven P, van Buchem MA, Elzinga BM, Rombouts SARB (2011) Beyond acute social stress: increased functional connectivity between amygdala and cortical midline structures. Neuroimage 57:1534–1541. 10.1016/j.neuroimage.2011.05.074 21664280

[B63] Verbruggen F, Logan GD (2009a) Proactive adjustments of response strategies in the stop-signal paradigm. J Exp Psychol Hum Percept Perform 35:835–854. 10.1037/a0012726 19485695 PMC2690716

[B64] Verbruggen F, Logan GD (2009b) Models of response inhibition in the stop-signal and stop-change paradigms. Neurosci Biobehav Rev 33:647–661. 10.1016/j.neubiorev.2008.08.014 18822313 PMC2696813

[B65] Verbruggen F, Aron AR, Band GP, Beste C, Bissett PG, Brockett AT, Brown JW, Chamberlain SR, Chambers CD, Colonius H, Colzato LS, Corneil BD, Coxon JP, Dupuis A, Eagle DM, Garavan H, Greenhouse I, Heathcote A, Huster RJ, Jahfari S, et al. (2019) A consensus guide to capturing the ability to inhibit actions and impulsive behaviors in the stop-signal task. Elife 8:e46323. 10.7554/eLife.46323 31033438 PMC6533084

[B66] Vink M, Kahn RS, Raemaekers M, van den Heuvel M, Boersma M, Ramsey NF (2005) Function of striatum beyond inhibition and execution of motor responses. Hum Brain Mapp 25:336–344. 10.1002/hbm.20111 15852388 PMC6871687

[B67] Vink M, Ramsey NF, Raemaekers M, Kahn RS (2006) Striatal dysfunction in schizophrenia and unaffected relatives. Biol Psychiatry 60:32–39. 10.1016/j.biopsych.2005.11.026 16603134

[B68] Westwater ML, Mancini F, Shapleske J, Serfontein J, Ernst M, Ziauddeen H, Fletcher PC (2020) Dissociable hormonal profiles for psychopathology and stress in anorexia and bulimia nervosa. Psychol Med. Advance online publication. Retrieved May 28, 2020. doi: 10.1017/s0033291720001440. 10.1017/s0033291720001440 PMC8640366 32460904

[B69] Wu M, Hartmann M, Skunde M, Herzog W, and Friederich H-C (2013) Inhibitory control in bulimic-type eating disorders: a systematic review and meta-analysis. PLoS One 8:e83412. 10.1371/journal.pone.0083412 24391763 PMC3877018

[B70] Yu J, Tseng P, Hung DL, Wu SW, Juan CH (2015) Brain stimulation improves cognitive control by modulating medial-frontal activity and preSMA-vmPFC functional connectivity. Hum Brain Mapp 36:4004–4015. 10.1002/hbm.22893 26248582 PMC6869595

[B71] Zandbelt BB, Vink M (2010) On the role of the striatum in response inhibition. PLoS One 5:e13848. 10.1371/journal.pone.0013848 21079814 PMC2973972

[B72] Zandbelt BB, Van Buuren M, Kahn RS, Vink M (2011) Reduced proactive inhibition in schizophrenia is related to corticostriatal dysfunction and poor working memory. Biol Psychiatry 70:1151–1158. 10.1016/j.biopsych.2011.07.028 21903198

[B73] Zandbelt BB, Bloemendaal M, Neggers SFW, Kahn RS, Vink M (2013) Expectations and violations: delineating the neural network of proactive inhibitory control. Hum Brain Mapp 34:2015–2024. 10.1002/hbm.22047 22359406 PMC6869973

