# Supplementary material for: Prefrontal Responses during Proactive and Reactive Inhibition Are Differentially Impacted by Stress in Anorexia and Bulimia Nervosa
Source: J Neurosci. 2021 May 19;41(20):4487–99. doi: 10.1523/JNEUROSCI.2853-20.2021 (PMC8152613; doi:10.1523/JNEUROSCI.2853-20.2021)
Supplement: Extended Data Figure 6-1 — Supplementary Figure 6-1. Download figure 6-1, DOCX file. [file ns-JN-RM-2853-20-s09.docx]

**EXTENDED DATA**

**Prefrontal responses during proactive and reactive inhibition are differentially impacted by stress in anorexia and bulimia nervosa**

Margaret L. Westwater, MPhil^1,2^, Flavia Mancini, PhD^3^, Adam X. Gorka, PhD^2^, Jane Shapleske, MD^4^, Jaco Serfontein, MD^4^, Christian Grillon, PhD^2^, Monique Ernst, MD, PhD^2^, Hisham Ziauddeen, MRCPsych, PhD^1,5^* & Paul C. Fletcher, MRCPsych, PhD^1,5^*

**Figure 1-1**. *Total plasma cortisol responses following acute stress versus a neutral task.* Total plasma cortisol is displayed as percentage change from baseline by group. The baseline sample was acquired pre-induction, and the second sample (+10 min) was acquired immediately following the induction. Remaining samples were acquired at 20 min. intervals. Full details of sample acquisition and analysis are reported in Westwater et al. (2020). Error bars = SEM.

**
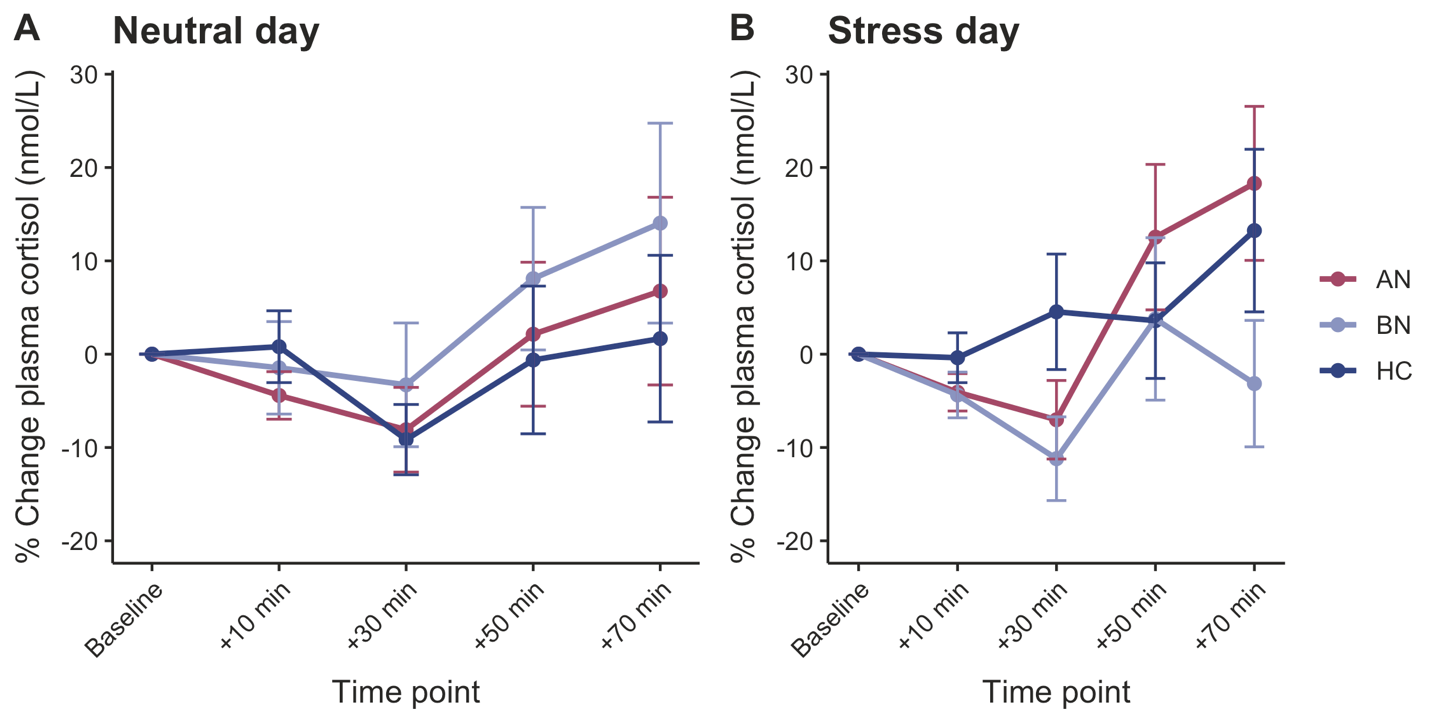
**

**Table 1-1.** *Psychotropic medication by patient group.*

| Medication | AN-BP | | BN | |
| --- | --- | --- | --- | --- |
|  | **# (%)** | **Dose (mg)** | **# (%)** | **Dose (mg)** |
| Amitriptyline | 1 (4.5) | 20 | - | - |
| Aripiprazole | 1 (4.5) | 5 | - | - |
| Bupropion | 1 (4.5) | 300 | - | - |
| Duloxetine | 1 (4.5) | 60 | - | - |
| Fluoxetine | 3 (13.6) | 40 – 60 | 4 (12.1) | 30 - 60 |
| Mirtazapine | - | - | 1 (3.0) | 15 |
| Olanzapine | 1 (4.5) | 5 | - | - |
| Sertraline | 2 (9.1) | 20 – 150 | 3 (9.1) | 100 |
| Venlafaxine | 1 (4.5) | 112.5 | 3 (9.1) | 150 – 300 |
| Zopiclone | - | - | 1 (3.0) | 3.75 |

**Note:** Several participants were prescribed more than one medication. Dose indicates mg/day.

**Figure 3-1.** Whole-brain fMRI responses to stop-signal probability by group

| Group | Side | Region | Peak MNI Coordinates | | | Size (voxels) | Z-statistic |
| --- | --- | --- | --- | --- | --- | --- | --- |
|  |  |  | X | Y | Z |  |  |
| AN | R | Inferior occipital gyrus | 34 | -81 | -6 | 511 | 7.25 |
|  | L | Inferior occipital gyrus | -35 | -96 | -6 | 461 | 7.13 |
|  | R | Middle insula | 40 | 15 | 6 | 174 | 6.03 |
|  | L | Supramarginal gyrus | -62 | -30 | 45 | 109 | 5.03 |
|  | R | Superior occipital gyrus | 22 | -78 | 45 | 71 | 4.58 |
|  | R | Precentral gyrus | 52 | 9 | 33 | 69 | 4.53 |
|  | R | Supramarginal gyrus | 61 | -33 | 51 | 39 | 4.54 |
|  | L | Middle insula | -35 | 18 | 12 | 27 | 4.88 |
|  | R | Cerebellar vermis | 4 | -72 | -9 | 24 | 4.06 |
|  | L | Middle frontal gyrus | -26 | -3 | 60 | 21 | 4.39 |
| BN | L | Inferior occipital gyrus | -35 | -99 | -9 | 365 | 6.78 |
|  | R | Inferior occipital gyrus | 34 | -93 | -6 | 380 | 8.01 |
|  | L | Postcentral gyrus | -20 | -45 | 75 | 19 | -4.13 |
| HC | L | Fusiform gyrus | -35 | -78 | -9 | 639 | 7.77 |
|  | R | Inferior occipital gyrus | 31 | -90 | -6 | 441 | 9.28 |
|  | R | Intraparietal sulcus | 31 | -78 | 45 | 94 | 4.61 |
|  | R | Precentral gyrus | 46 | 9 | 33 | 60 | 5.08 |
|  | L | Rostral middle frontal gyrus | -20 | 57 | 36 | 52 | -4.35 |
|  | L | Inferior frontal gyrus (pars orbitalis) | -44 | 27 | -9 | 51 | -5.32 |
|  | L | Middle frontal gyrus | -44 | 24 | 51 | 45 | -4.58 |
|  | R | Postcentral gyrus | 61 | -24 | 39 | 31 | 5.17 |
|  | L | Supramarginal gyrus | -62 | -30 | 39 | 28 | 4.35 |
|  | R | SMA | 4 | 6 | 60 | 24 | 4.96 |
|  | L | Superior frontal gyrus | -5 | 42 | 57 | 23 | -4.17 |

Note: Clusters were defined at a cluster-defining threshold of p < .001 and FWE-corrected at p < .05 (kE = 18.8 voxels). MNI coordinates represent the peak voxel within each cluster.

**Figure 3-2.** Whole-brain fMRI responses to reaction time amplitude modulator by group

| Group | Side | Region | Peak MNI Coordinates | | | Size (voxels) | Z-statistic |
| --- | --- | --- | --- | --- | --- | --- | --- |
|  |  |  | X | Y | Z |  |  |
| AN | L | Primary motor cortex | -41 | -27 | 60 | 1846 | 7.80 |
|  | R | Inferior occipital gyrus | 34 | -93 | 3 | 561 | 6.97 |
|  | R | Superior parietal lobule | 16 | -60 | 72 | 352 | 6.26 |
|  | R | SMA | 1 | -9 | 57 | 336 | 7.84 |
|  | R | Cerebellar vermis | 4 | -72 | -30 | 300 | 6.20 |
|  | R | Superior frontal gyrus | 25 | -12 | 69 | 170 | 7.11 |
|  | L | Anterior cingulate cortex | -14 | 42 | 12 | 157 | -4.98 |
|  | L | Calcarine sulcus | -14 | -81 | 15 | 142 | 5.34 |
|  | R | Supramarginal gyrus | 67 | -33 | 33 | 108 | 4.87 |
|  | L | Middle cingulate cortex | -2 | -39 | 45 | 108 | -4.86 |
|  | L | Angular gyrus | -44 | -72 | 57 | 50 | -4.39 |
|  | L | Supramarginal gyrus | -50 | -30 | 21 | 41 | 5.00 |
|  | L | Thalamus (prefrontal) | -14 | -24 | 12 | 30 | 4.54 |
|  | L | Middle frontal gyrus | -38 | 6 | 63 | 29 | -4.28 |
|  | R | Frontal opercular | 49 | 0 | 12 | 24 | 4.57 |
|  | R | Cerebellar cortex | 16 | -96 | -30 | 19 | -4.98 |
|  | R | Caudate nucleus | 7 | 6 | 0 | 19 | -3.90 |
| BN | L | Precuneus | -11 | -66 | 69 | 1688 | 8.65 |
|  | R | Precuneus | 10 | -60 | 72 | 818 | 8.74 |
|  | L | SMA | -2 | -6 | 60 | 382 | 7.50 |
|  | R | Medial frontal gyrus | 7 | 30 | -9 | 224 | -5.37 |
|  | R | Cerebellar cortex | 19 | -63 | -18 | 222 | 4.97 |
|  | R | Middle temporal gyrus | 52 | -66 | 25 | 199 | 4.93 |
|  | R | Superior frontal gyrus | 28 | -9 | 72 | 184 | 5.86 |
|  | L | Middle occipital gyrus | -41 | -81 | 45 | 172 | -5.14 |
|  | R | Supramarginal gyrus | 61 | -36 | 45 | 147 | 4.70 |
|  | L | Caudate nucleus | -5 | 6 | 3 | 126 | -5.54 |
|  | L | Inferior frontal gyrus (pars orbitalis) | -53 | 24 | 0 | 111 | -4.82 |
|  | L | Superior frontal gyrus | -11 | 57 | 42 | 85 | -4.51 |
|  | L | Middle occipital gyrus | -50 | -87 | 6 | 75 | 5.00 |
|  | L | Middle cingulate cortex | -5 | -42 | 45 | 73 | -5.04 |
|  | L | Middle frontal gyrus | -23 | 18 | 57 | 71 | -4.86 |
|  | R | Cerebellar cortex | 46 | -75 | -30 | 67 | -6.59 |
|  | L | Lingual gyrus | -23 | -96 | -9 | 64 | 4.80 |
|  | L | Cerebellar cortex | -35 | -48 | -27 | 62 | 5.09 |
|  | R | Cerebellar cortex | 10 | -87 | -21 | 54 | -5.03 |
|  | L | Cuneus | -20 | -78 | 9 | 34 | 4.88 |
|  | L | Parieto-occipital sulcus | -11 | -60 | 21 | 30 | -5.04 |
|  | L | Superior frontal gyrus | -23 | 57 | 9 | 29 | -4.56 |
|  | L | Orbitofrontal cortex | -26 | 21 | -15 | 27 | -4.74 |
|  | L | Rostral middle frontal | -56 | 18 | 36 | 24 | -4.66 |
|  | L | Caudal middle frontal | -41 | 12 | 39 | 21 | -4.68 |
|  | L | Inferior temporal gyrus | -56 | -51 | -9 | 20 | -5.34 |
| HC | L | Precentral gyrus | -30 | -7 | 66 | 2854 | 9.21 |
|  | R | Precuneus | 7 | -63 | 72 | 1601 | 8.43 |
|  | R | Cerebellar vermis (VII) | 4 | -78 | -27 | 471 | 6.75 |
|  | R | Anterior cingulate cortex | 4 | 36 | 6 | 191 | -5.56 |
|  | L | Middle occipital gyrus | -38 | -93 | 0 | 130 | 4.66 |
|  | L | Cerebellar vermis (VI) | -35 | -48 | -27 | 111 | 5.66 |
|  | L | Putamen | -14 | -3 | -6 | 90 | -4.69 |
|  | L | Middle orbital gyrus | -5 | 54 | 3 | 75 | -5.38 |
|  | L | Thalamus (prefrontal) | -14 | -24 | 12 | 64 | 7.23 |
|  | R | Thalamus (prefrontal) | 10 | -21 | 12 | 63 | 6.09 |
|  | R | Inferior occipital gyrus | 22 | -93 | -3 | 55 | 5.56 |
|  | L | Calcarine sulcus | -11 | -60 | 12 | 33 | -4.74 |
|  | R | Thalamus/red nucleus | 7 | -24 | -6 | 22 | 4.83 |
|  | R | Fusiform gyrus | 25 | -87 | -12 | 21 | 4.75 |
|  | L | Medial frontal gyrus | -2 | 57 | 21 | 20 | -4.16 |
|  | L | Angular gyrus | -38 | -78 | 48 | 20 | -3.90 |
|  | R | Prostriate area | 28 | -54 | 18 | 19 | -4.14 |

Note: Clusters were defined at a cluster-defining threshold of p < .001 and FWE-corrected at p < .05 (kE = 18.8 voxels). MNI coordinates represent the peak voxel within each cluster.

**Figure 3-3.** Whole-brain fMRI responses during reactive inhibition (Successful Stop > Go-signal 0%) by group

| Group | Side | Region | Peak MNI Coordinates | | | Size (voxels) | Z-statistic |
| --- | --- | --- | --- | --- | --- | --- | --- |
|  |  |  | X | Y | Z |  |  |
| AN | L | Postcentral gyrus | -32 | -36 | 69 | 6396 | -10.77 |
|  | R | Inferior frontal gyrus (pars opercularis) | 40 | 12 | 9 | 3914 | 10.98 |
|  | R | Medial orbitofrontal | 7 | 33 | -9 | 2828 | -9.11 |
|  | R | Middle occipital gyrus | 31 | -72 | 39 | 1951 | 8.03 |
|  | L | Middle occipital gyrus | -44 | -81 | 42 | 335 | -6.82 |
|  | L | Inferior parietal lobule | -29 | -63 | 54 | 250 | 7.28 |
|  | L | Fusiform gyrus | -35 | -63 | -9 | 217 | 6.15 |
|  | L | Cerebellar cortex (VI) | -35 | -57 | -30 | 200 | 7.30 |
|  | R | Cerebellar cortex (VIIa) | 34 | -84 | -30 | 190 | -6.91 |
|  | R | Superior frontal gyrus | 22 | 30 | 54 | 172 | -7.69 |
|  | L | Rostral middle frontal gyrus | -38 | 42 | 36 | 116 | 5.69 |
|  | R | Angular gyrus | 49 | -72 | 39 | 114 | -6.12 |
|  | R | Middle cingulate cortex | 7 | -24 | 33 | 81 | 5.89 |
|  | L | Supramarginal gyrus | -65 | -33 | 30 | 74 | 6.21 |
|  | R | Inferior occipital gyrus | 25 | -105 | 3 | 70 | -6.67 |
|  | R | Superior temporal gyrus | 49 | -30 | 3 | 65 | 5.95 |
|  | L | Middle occipital gyrus | -29 | -102 | 0 | 56 | -5.74 |
|  | L | Cerebellar cortex (VIIa) | -17 | -93 | -33 | 45 | -5.29 |
|  | R | Lateral orbitofrontal | 37 | 30 | -12 | 40 | -6.98 |
|  | R | Cerebellar cortex (VI) | 34 | -57 | -27 | 36 | 6.02 |
|  | R | Caudate nucleus | 16 | 15 | 21 | 27 | -5.32 |
| BN | L | Calcarine sulcus | -8 | -69 | -21 | 6350 | -10.26 |
|  | R | Inferior occipital gyrus | 4 | 9 | 54 | 5381 | 12.34 |
|  | R | Intraparietal sulcus | 31 | -75 | 36 | 3430 | 10.52 |
|  | L | Medial orbitofrontal | -8 | 39 | -9 | 2572 | -10.26 |
|  | L | Cerebellar cortex (VI) | -32 | -60 | -27 | 1887 | 9.26 |
|  | L | Angular gyrus | -53 | -72 | 33 | 550 | -8.69 |
|  | R | Cerebellum | 16 | -93 | -33 | 227 | -8.85 |
|  | R | Superior frontal gyrus | 19 | 33 | 60 | 181 | -6.82 |
|  | L | Rostral middle frontal gyrus | -35 | 42 | 30 | 172 | 6.27 |
|  | R | Angular gyrus | 52 | -72 | 36 | 138 | -6.89 |
|  | R | Superior temporal gyrus | 49 | -27 | 0 | 92 | 6.44 |
|  | L | Cerebellar cortex (VIIa) | -17 | -93 | -33 | 85 | -6.89 |
|  | L | Middle occipital gyrus | -23 | -108 | 3 | 67 | -6.31 |
|  | R | Inferior occipital gyurs | 28 | -105 | 0 | 64 | -8.25 |
|  | R | Caudate | 19 | -18 | 36 | 57 | -6.21 |
|  | R | Prostriate area | 31 | -54 | 6 | 48 | -6.83 |
|  | R | Inferior frontal gyrus (pars orbitalis) | 37 | 30 | -12 | 37 | -6.12 |
|  | R | Dorsolateral putamen | 28 | -24 | 9 | 28 | 5.40 |
| HC | L | Calcarine sulcus | -8 | -69 | 21 | 11370 | -10.77 |
|  | R | Inferior frontal gyrus (pars opercularis) | 40 | 15 | 9 | 4124 | 12.03 |
|  | R | Middle occipital sulcus | 34 | -75 | 36 | 2264 | 9.69 |
|  | L | Insula | -32 | 15 | 9 | 714 | 12.14 |
|  | L | Superior parietal lobule | -26 | -69 | 57 | 563 | 7.13 |
|  | L | Fusiform gyrus | -35 | -63 | -6 | 508 | 8.53 |
|  | L | Middle cingulate cortex | -8 | -30 | 33 | 237 | 7.92 |
|  | L | Cerebellar cortex (VIIa) | -17 | -93 | -33 | 194 | -8.09 |
|  | R | Superior frontal gyrus | 22 | 30 | 54 | 146 | -6.80 |
|  | L | Cerebellar cortex (VI) | -32 | -57 | -30 | 123 | 8.20 |
|  | R | Angular gyrus | 49 | -72 | 39 | 101 | -6.57 |
|  | L | Middle frontal gyrus | -35 | 42 | 30 | 100 | 6.38 |
|  | L | Cerebellar cortex | -8 | -78 | -18 | 75 | 6.28 |
|  | R | Superior temporal gyrus | 49 | -30 | 0 | 59 | 5.24 |
|  | R | Dorsal caudate | 16 | 0 | 33 | 42 | -6.21 |
|  | R | Cerebellar cortex (VI) | 34 | -57 | -27 | 28 | 5.47 |
|  | R | Inferior frontal gyrus (pars orbitalis) | 37 | 30 | -12 | 28 | -4.94 |
|  | R | Caudate | 19 | -18 | 36 | 28 | -6.11 |

Note: Clusters were defined at a cluster-defining threshold of p < .001 and FWE-corrected at p < .05 (kE = 18.8 voxels). MNI coordinates represent the peak voxel within each cluster.

**Figure 3-4.** Whole-brain fMRI responses during reactive inhibition (Successful Stop > Failed Stop) by group

| Group | Side | Region | Peak MNI Coordinates | | | Size (voxels) | Z-statistic |
| --- | --- | --- | --- | --- | --- | --- | --- |
|  |  |  | X | Y | Z |  |  |
| AN | R | Cerebellum (IV-V) | 10 | -60 | -18 | 3659 | -9.24 |
|  | L | Postcentral gyrus | -44 | -24 | 57 | 1154 | -9.29 |
|  | R | Middle cingulate cortex | 1 | 18 | 39 | 1104 | -7.96 |
|  | L | Inferior frontal gyrus (pars opercularis) | -53 | 9 | 3 | 434 | -8.43 |
|  | R | Inferior frontal gyrus (pars opercularis) | 52 | 9 | 0 | 393 | -8.20 |
|  | R | SMA | 10 | 6 | 72 | 273 | -6.12 |
|  | R | Middle temporal gyrus | 58 | -48 | 15 | 141 | -4.62 |
|  | R | Rostral middle frontal | 28 | 54 | 30 | 124 | -5.08 |
|  | R | Middle temporal gyrus | 46 | -75 | 18 | 115 | -6.03 |
|  | L | Middle frontal gyrus | -32 | 51 | 27 | 109 | -4.87 |
|  | L | Superior parietal lobule | -32 | -69 | 63 | 70 | -4.85 |
|  | R | Cerebellar vermis | 4 | -75 | -30 | 45 | -6.85 |
|  | R | Primary sensory cortex | 19 | -33 | 69 | 43 | -4.61 |
|  | R | Precentral gyrus | 49 | 3 | 54 | 42 | -4.60 |
|  | R | Inferior parietal lobule | 49 | -51 | 60 | 29 | -4.91 |
|  | R | Superior parietal lobule | 22 | -78 | 57 | 26 | -4.06 |
|  | L | Posterior insula | -38 | -15 | -3 | 23 | -4.61 |
|  | R | Putamen | 28 | -6 | 6 | 22 | 4.24 |
|  | L | Middle temporal gyrus | -59 | -60 | 18 | 21 | -4.10 |
|  | L | Caudate nucleus | -8 | 18 | 6 | 20 | -4.33 |
|  | R | Postcentral gyrus | 64 | -18 | 33 | 20 | -4.14 |
|  | R | Precuneus | 10 | -75 | 66 | 20 | -4.87 |
| BN | L | Middle occipital gyrus | -32 | -87 | 24 | 12819 | -11.61 |
|  | R | Inferior occipital gyrus | 34 | -93 | -6 | 725 | -7.60 |
|  | L | Insula | -32 | 15 | -6 | 608 | -7.97 |
|  | R | Superior frontal gyrus | 22 | 51 | 30 | 164 | -6.54 |
|  | L | Middle frontal gyrus | -32 | 45 | 24 | 97 | -5.18 |
|  | R | Precentral gyrus | 25 | -33 | 78 | 83 | -5.07 |
|  | L | Putamen | -29 | -3 | 6 | 71 | 5.84 |
|  | R | Putamen | 25 | 3 | 0 | 48 | 5.27 |
|  | L | Angular gyrus | -50 | -72 | 54 | 43 | 5.73 |
|  | L | Middle frontal gyrus | -26 | 24 | 63 | 34 | 4.31 |
|  | R | Posterior insula | 40 | -15 | 3 | 31 | -4.67 |
|  | R | Inferior temporal gyrus | 46 | -6 | -27 | 30 | -4.71 |
|  | L | Middle temporal gyrus | -62 | -21 | 6 | 28 | 4.39 |
|  | L | Inferior frontal gyrus (pars orbitalis) | -41 | 42 | -6 | 24 | 4.75 |
| HC | L | Anterior cingulate cortex | -5 | 18 | 36 | 11517 | -10.05 |
|  | R | Inferior frontal gyrus (pars opercularis) | 49 | 9 | 6 | 402 | -8.04 |
|  | R | Precentral gyrus | 49 | 3 | 54 | 273 | -6.56 |
|  | L | Inferior parietal lobule | -50 | -66 | 54 | 50 | 5.12 |
|  | L | Middle temporal gyrus | 49 | -24 | -6 | 39 | -4.50 |
|  | L | Para-insular area | -41 | -18 | -3 | 29 | -5.85 |
|  | R | Middle frontal gyrus | 31 | 36 | 30 | 26 | -4.08 |
|  | L | Medial frontal gyrus | -8 | 33 | -9 | 23 | 5.39 |
|  | L | Middle frontal gyrus | -29 | 42 | 33 | 23 | -5.03 |
|  | L | Middle frontal gyrus | -32 | 21 | 57 | 22 | 4.11 |
|  | R | Superior frontal gyrus | 34 | 54 | 6 | 21 | 3.90 |

Note: Clusters were defined at a cluster-defining threshold of p < .001 and FWE-corrected at p < .05 (kE = 18.8 voxels). MNI coordinates represent the peak voxel within each cluster.

**Figure 4-1.** Whole-brain linear mixed-effects analysis of proactive inhibition

| Effect | Direction | Side | Region | Peak MNI Coordinates | | | Size (voxels) | F-statistic | Z-statistic |
| --- | --- | --- | --- | --- | --- | --- | --- | --- | --- |
|  |  |  |  | X | Y | Z |  |  |  |
| *RT modulator* | | | |  |  |  |  |  |  |
| Time | Post < Pre | L | SMA | -5 | 20 | 65 | 35 | 21.58 | -4.22 |
| *Stop-signal probability modulator* | | | |  |  |  |  |  |  |
| Group X Condition X Time | BN > AN X stress > neutral X post > pre | R | Superior frontal gyrus | 22 | 54 | 36 | 19 | 10.77 | 4.52 |
| Group | BN > HC | L | Superior frontal gyrus | -23 | 33 | 54 | 25 | 11.88 | 4.58 |
| Time | Post < Pre | L | Inferior occipital gyrus | -41 | -84 | -6 | 618 | 74.34 | -8.62 |
|  | Post < Pre | R | Inferior occipital gyrus | 37 | -87 | -6 | 430 | 58.05 | -7.62 |
|  | Post < Pre | R | Inferior frontal gyrus (pars triangularis) | 43 | 24 | 30 | 190 | 35.98 | -6.00 |
|  | Post < Pre | R | Cerebellar vermis | 1 | -45 | 6 | 170 | 37.29 | -6.11 |
|  | Post < Pre | R | Inferior parietal lobule | 49 | -36 | 54 | 106 | 24.81 | -4.98 |
|  | Post < Pre | L | Superior parietal lobule | -23 | -72 | 45 | 96 | 28.95 | -5.38 |
|  | Post < Pre | L | Precentral gyrus | -47 | -3 | 42 | 70 | 32.17 | -5.67 |
|  | Post < Pre | L | Cingulate gyrus | -23 | -48 | 24 | 66 | 27.36 | -5.23 |
|  | Post < Pre | L | Inferior frontal gyrus (pars triangularis) | -53 | 33 | 27 | 58 | 30.08 | -5.48 |
|  | Post < Pre | R | Caudate | 25 | -45 | 21 | 58 | 28.08 | -5.30 |
|  | Post < Pre | L | Anterior insula | -32 | 21 | 0 | 56 | 26.08 | -5.11 |
|  | Post < Pre | R | Anterior insula | 37 | 21 | 3 | 53 | 21.13 | -4.60 |
|  | Post < Pre | R | Intraparietal sulcus | 31 | -72 | 36 | 52 | 28.33 | -5.32 |
|  | Post < Pre | L | Inferior parietal lobule | -56 | -39 | 54 | 50 | 22.22 | -4.71 |
|  | Post > Pre | R | Cuneus | 10 | -93 | 39 | 45 | 25.59 | 5.06 |
|  | Post < Pre | R | Supplementary motor area | 7 | 15 | 51 | 45 | 33.49 | -5.79 |
|  | Post < Pre | R | Middle occipital gyrus | 40 | -90 | 15 | 44 | 27.02 | -5.20 |
|  | Post < Pre | L | Middle occipital gyrus | -35 | -93 | 18 | 37 | 28.29 | -5.32 |
|  | Post < Pre | L | Thalamus (temporal) | -5 | -9 | 9 | 26 | 25.74 | -5.07 |
|  | -- | L | Thalamus (proper) | -2 | -21 | 18 | 26 | 20.18 | -- |
|  | Post > Pre | L | Angular gyrus | -56 | -66 | 51 | 24 | 17.33 | 4.16 |
|  | Post < Pre | L | Middle frontal gyrus | -26 | -6 | 57 | 24 | 21.08 | -4.59 |
|  | Post < Pre | L | Middle temporal gyrus | -56 | -51 | 18 | 19 | 23.19 | -4.89 |
|  | Post < Pre | L | Inferior parietal lobule | -35 | -51 | 48 | 19 | 18.16 | -4.26 |

Note: RT modulator = parametric effect of reaction time during non-0% Go trials contrast; Stop-signal probability modulator = parametric effect of stop-signal probability during non-0% Go trials contrast. Clusters were identified at a cluster-defining threshold of p < .001 and FWE-corrected at p < .05 (kE = 18.8 voxels). For completeness, both F- and Z-statistics are reported for each effect. Cluster size correspond to the F-statistic map. MNI coordinates represent the peak voxel within each cluster.

**Figure 5-1.** Whole-brain linear mixed-effects analysis of reactive inhibition

| Effect | Direction | Side | Region | Peak MNI Coordinates | | | Size (voxels) | F-statistic | Z-statistic |
| --- | --- | --- | --- | --- | --- | --- | --- | --- | --- |
|  |  |  |  | X | Y | Z |  |  |  |
| *Successful Stop > Failed Stop* | | | |  |  |  |  |  |  |
| Group X Condition X Time | AN > HC X  Stress > Neutral X Post > Pre | R | Medial frontal gyrus | 4 | 45 | -9 | 30 | 12.35 | -4.19 |
| Condition | Stress < Neutral | L | Precentral gyrus | -59 | -3 | 33 | 24 | 16.16 | -4.02 |
| Time | Post < Pre | L | Middle temporal gyrus | -59 | -60 | 18 | 37 | 14.90 | -3.86 |
|  | Post < Pre | L | Thalamus (prefrontal) | -8 | -18 | 12 | 21 | 17.68 | -4.21 |
|  | Post < Pre | L | Posterior insula | -44 | -3 | 0 | 21 | 16.33 | -4.04 |
|  | Post < Pre | L | Superior occipital gyrus | -20 | -78 | 33 | 19 | 15.53 | -3.94 |
| *Successful Stop > Go 0%* | | | |  |  |  |  |  |  |
| Time | Post < Pre | L | Superior medial gyrus, extending to SMA | 1 | 24 | 60 | 341 | 40.33 | -6.45 |
|  | Post < Pre | R | Middle frontal gyrus | 43 | 12 | 57 | 310 | 30.47 | -5.52 |
|  | Post < Pre | R | Angular gyrus | 52 | -57 | 39 | 166 | 30.97 | -5.56 |
|  | Post < Pre | R | Inferior frontal gyrus (pars opercularis) | 43 | 18 | -6 | 142 | 32.07 | -5.66 |
|  | Post > Pre | L | Primary sensory cortex | -41 | -42 | 66 | 120 | 21.55 | 4.64 |
|  | Post < Pre | L | Inferior frontal gyrus (pars opercularis) | -35 | 15 | -9 | 68 | 39.00 | -6.25 |
|  | Post < Pre | L | Cerebellum | -26 | -84 | -24 | 63 | 20.14 | -4.49 |
|  | Post < Pre | R | Superior frontal gyrus | 25 | 54 | 15 | 63 | 41.72 | -6.46 |
|  | Post < Pre | R | Middle temporal gyrus | 64 | -45 | 0 | 51 | 24.26 | -4.93 |
|  | Post > Pre | R | Superior parietal lobule | 16 | -57 | 75 | 30 | 17.98 | 4.24 |
|  | Post < Pre | L | Inferior temporal gyrus | -47 | -60 | -9 | 24 | 18.48 | -4.30 |
|  | Post < Pre | L | Precentral gyrus | -56 | 6 | 45 | 25 | 20.13 | -4.49 |
|  | Post < Pre | R | Caudate nucleus | 4 | -3 | 12 | 25 | 23.93 | -4.89 |
|  | Post < Pre | L | Premotor cortex | -44 | -3 | 45 | 25 | 17.48 | -4.18 |
|  | Post > Pre | R | Precentral gyrus | 37 | -24 | 72 | 21 | 17.28 | 4.16 |
|  | Post > Pre | L | Postcentral gyrus | -53 | -21 | 60 | 19 | 17.80 | 4.22 |

Note: Clusters were defined at a cluster-defining threshold of p < .001 and FWE-corrected at p < .05 (kE = 18.8 voxels). Cluster size was determined from the F-statistic map. MNI coordinates represent the peak voxel within each cluster.

**Figure 6-1.** *Ad libitum meal contents and macronutrient information*

| **Item** | **Amount available (g)** | **Fat (g) in 100g** | **Total fat available (g)** | **CHO(g) in 100g** | **Total CHO available (g)** | **Protein (g) in 100g** | **Total protein available (g)** | **Total energy provided (kcal)** |
| --- | --- | --- | --- | --- | --- | --- | --- | --- |
| Semi-Skimmed Milk | 254.0 | 1.8 | 4.6 | 4.8 | 12.2 | 3.6 | 9.2 | 127.08 |
| Tripoca Trop 50 Orange Juice | 254.0 | 0.0 | 0.0 | 3.9 | 9.9 | 0.3 | 0.8 | 53.37 |
| The Food Doctor High Fibre and Cereal Pitta (x2) | 102.0 | 1.7 | 1.7 | 24.2 | 24.6 | 5.9 | 6.0 | 145.38 |
| Tesco Reduced Fat Sour Cream Dip | 85.0 | 14.6 | 12.4 | 7.3 | 6.2 | 3.9 | 3.3 | 149.10 |
| Tesco Reduced Fat Hummus | 85.0 | 10.5 | 8.9 | 11.0 | 9.3 | 6.4 | 5.4 | 152.49 |
| Doritos | 42.0 | 14.0 | 5.9 | 31.0 | 13.1 | 3.4 | 1.4 | 115.64 |
| Walkers Baked Ready Salted Crisps | 42.0 | 13.5 | 5.7 | 69.0 | 29.2 | 6.6 | 2.8 | 184.68 |
| Carrot Batons | 68.0 | 0.3 | 0.2 | 7.7 | 5.2 | 0.6 | 0.4 | 28.47 |
| Seedless Grapes | 85.0 | 0.1 | 0.1 | 15.4 | 13.1 | 0.4 | 0.3 | 55.91 |
| Foxes Shortbread Viennese Dark Chocolate Biscuits | 42.0 | 29.0 | 12.3 | 55.0 | 23.3 | 5.4 | 2.3 | 221.96 |
| Oreo Biscuits | 56.0 | 20.0 | 11.2 | 69.0 | 38.6 | 5.0 | 2.8 | 268.39 |
| Rice Krispie Squares (x2) | 47.0 | 12.0 | 5.7 | 76.0 | 36.1 | 3.0 | 1.4 | 201.15 |
| Be Good To Yourself Vegetable Pasta | 424.0 | 1.4 | 5.9 | 15.2 | 64.4 | 2.8 | 11.9 | 376.99 |
| Tesco Breaded Chicken Goujons | 169.0 | 13.9 | 23.6 | 19.3 | 32.7 | 18.4 | 31.2 | 469.34 |
| Tesco Stonebaked Four Cheese Pizza | 280.0 | 9.5 | 26.6 | 27.2 | 76.0 | 12.9 | 36.1 | 701.72 |
| Tesco Chocolate Brownie Traybake (serve whole traybake) | 188.0 | 20.0 | 37.6 | 54.8 | 103.1 | 6.1 | 11.5 | 808.71 |
| **TOTAL** | 2223.0 | - | 162.3 | - | 497.0 | - | 126.7 | 4060.4 |

Note: CHO = carbohydrate. Contents of the ad libitum meal were the same across Days 1 and 2.
